# Transcriptome analysis of porcine *M. semimembranosus* divergent in intramuscular fat as a consequence of dietary protein restriction

**DOI:** 10.1186/1471-2164-14-453

**Published:** 2013-07-06

**Authors:** Ruth M Hamill, Ozlem Aslan, Anne M Mullen, John V O’Doherty, Jean McBryan, Dermot G Morris, Torres Sweeney

**Affiliations:** 1 Teagasc Food Research Centre, Ashtown, Dublin 15, Ireland; 2 UCD School of Agriculture and Food Science, Dublin 4, Ireland; 3 Animal and Bioscience Research Department, Animal & Grassland Research and Innovation Centre, Teagasc, Athenry, Co. Galway, Ireland; 4 UCD School of Veterinary Medicine, Belfield, Dublin 4, Ireland

## Abstract

**Background:**

Intramuscular fat (IMF) content is positively correlated with aspects of pork palatability, including flavour, juiciness and overall acceptability. The ratio of energy to protein in the finishing diet of growing pigs can impact on IMF content with consequences for pork quality. The objective of this study was to compare gene expression profiles of *Musculus semimembranosus* (SM) of animals divergent for IMF as a consequence of protein dietary restriction in an isocaloric diet. The animal model was derived through the imposition of low or high protein diets during the finisher stage in Duroc gilts. RNA was extracted from *post mortem* SM tissue, processed and hybridised to Affymetrix porcine GeneChip® arrays.

**Results:**

IMF content of SM muscle was increased on the low protein diet (3.60 ± 0.38% versus 1.92 ± 0.35%). Backfat depth was also greater in animals on the low protein diet, and average daily gain and feed conversion ratio were lower, but muscle depth, protein content and moisture content were not affected. A total of 542 annotated genes were differentially expressed (DE) between animals on low and high protein diets, with 351 down-regulated and 191 up-regulated on the low protein diet. Transcript differences were validated for a subset of DE genes by qPCR. Alterations in functions related to cell cycle, muscle growth, extracellular matrix organisation, collagen development, lipogenesis and lipolysis, were observed. Expression of adipokines including *LEP*, *TNFα* and *HIF1α* were increased and the hypoxic stress response was induced. Many of the identified transcriptomic responses have also been observed in genetic and fetal programming models of differential IMF accumulation, indicating they may be robust biological indicators of IMF content.

**Conclusion:**

An extensive perturbation of overall energy metabolism in muscle occurs in response to protein restriction. A low protein diet can modulate IMF content of the SM by altering gene pathways involved in lipid biosynthesis and degradation; however this nutritional challenge negatively impacts protein synthesis pathways, with potential consequences for growth.

## Background

Intra-muscular fat (IMF), also known as marbling fat, is an adipocyte depot located within perimysial connective tissues alongside myofibres [1]. Intramuscular fat is a late-developing depot and intramuscular adipocytes have been shown to be metabolically distinct from adipocytes in other depots, such as subcutaneous fat, as reviewed by Hausman *et al.*[1]. IMF is very important in defining pork quality from a sensory perspective, since it is positively correlated with flavour, juiciness, tenderness/firmness and overall acceptability of pork [2-4]. Furthermore, IMF also has an important influence on the sensory characteristics of dry-cured ham, an economically significant processed product [5]. Some authors propose a minimum threshold of 1.5% IMF for acceptable eating quality in fresh pork [3], while other studies suggest that levels of 2 to 3% IMF are required to ensure desirable eating quality [6]. Currently, IMF levels in the majority of modern commercial breeds are typically less than 1.5% [7,8]. While this is largely due to genetic selection for lean growth, carcass yield, carcass composition and meat quality traits including IMF are influenced by an interaction between genotype and environmental factors such as level of nutrition [9-11]. An improved ability to manipulate IMF deposition could provide a basis to enhance uniformity in desirable eating quality characteristics in commercial production, but a more complete understanding of porcine muscle biology in relation to intramuscular fat deposition, in particular in the more commercially relevant muscles, is first required.

Dietary protein restriction during the growth/finisher phase in pigs, relative to overall energy content in the diet, is a nutritional strategy which has several profound effects on the pig, including reduced lean deposition, increased fat deposition throughout the carcass, and in particular, in the economically significant depot of IMF [12-15]. For example, feeding pigs a 15% reduced lysine:energy diet significantly increased IMF levels and furthermore improved juiciness, tenderness and overall acceptability scores by sensory panellists [12,16]. While extreme restriction strategies have also been shown to impair growth, limiting their relevance to the commercial setting, the generation of muscle displaying divergent IMF through such a dietary intervention provides a valuable resource to explore the physiology of this key eating quality trait.

Identification of gene pathways/networks whose expression is altered in response to nutritional modulation would provide insight into the physiological/biochemical processes important for muscle development and help to uncover the mechanisms involved in IMF deposition in porcine muscle [15,17]. The Duroc breed is characterised by moderate levels of IMF [18] which is more easily manipulated compared to other commercial breeds and it has been identified as a useful model for understanding the physiological basis for muscle lipid metabolism [19]. Within a carcass there are a number of muscles which exhibit favourable quality characteristics and command relatively high value. The *Musculus semimembranosus* (SM) is one such muscle that is particularly relevant to ham production. Hence, the objective of this study was to compare the phenotypic and transcriptomic response between Duroc pigs maintained on low and high protein diets during the finisher phase.

## Results and discussion

### Phenotypic response to dietary protein restriction

Although intakes did not differ significantly between animals on the low protein (LP) and high protein (HP) diets, average daily gain and feed conversion ratio were significantly lower in the animals on the LP diet (*P* < 0.001) (Table 1). While carcass weight did not differ significantly at slaughter, there was a tendency towards reduced carcass weight in animals on the LP diet (*P* < 0.1). Muscle depth did not differ between dietary treatments. However, fat depth measured between the 3rd and 4th rib was greater (*P* = 0.032) following the LP diet (Table 1). Composition analysis indicated that there was no effect of treatment on protein or moisture content in the muscle (*P* > 0.05). The LP and HP diets did, however, have a significant effect on fat content in the muscle: SM muscle derived from animals on the LP diet had almost twice as much IMF (Table 1) as muscle from the HP diet (3.60 ± 0.38% versus 1.92 ± 0.35%, *P <* 0.001). Our results concur with those of D’Souza et al. [20] and Bidner et al. [21] who also found a negative relationship with growth, as well as a positive increment in IMF deposition following protein restriction during growth. The two levels of IMF generated lie each side of the 2% IMF threshold for ‘desirable eating quality’ as suggested by Devol et al. [6] and thus provide a useful model for exploration of the molecular basis of eating quality.

**Table 1:** Animal performance, carcass and meat quality on dietary treatments

**Traits**	**LP diet (n = 5)**	**HP diet (n = 6)**	***P-value***
	**Mean ± S.E.**	**Mean ± S.E.**	
Initial weight (kg)	45.75 ± 2.85	46.50 ± 1.89	0.83
Feed intake (g/day)	1758.5 ± 127.2	2013.1 ± 107.0	0.170
Feed conversion ratio	3.40 ± 0.10	2.41 ± 0.11	*<0.001*
Average daily gain (kg)	0.52 ± 0.03	0.83 ± 0.03	*<0.001*
Carcass weight (kg)	61.62 ± 11.08	71.56 ± 5.85	0.088
Fat depth ¾ rib (mm)	9.55 ± 2.21	6.79 ± 1.39	*0.032*
Loin depth (mm)	54.24 ± 10.58	59.76 ± 5.24	0.29
IMF (%) SM day 2	3.60 ± 0.38	1.92 ± 0.35	*<0.001*
Protein (%) SM day 2	21.26 ± 0.36	22.19 ± 0.33	0.45
Moisture (%) SM day 2	73.48 ± 0.41	74.11 ± 0.38	0.93

SM: *M*. *semimembranosus* muscle.

### Identification and functional characterisation of differentially expressed genes in SM muscle associated with dietary protein restriction

**Figure 1:**
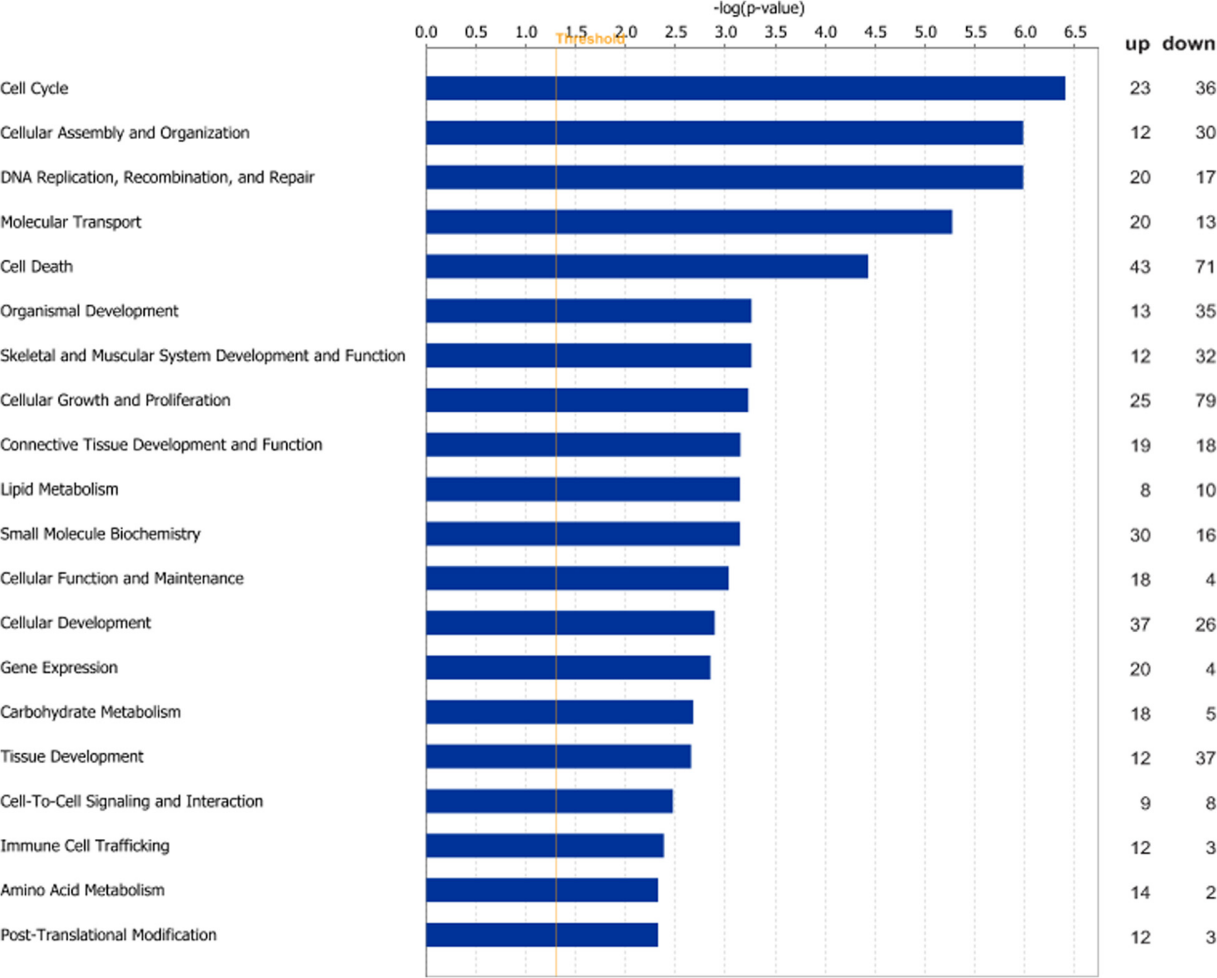
**IPA molecular and cellular functions significantly over-represented among differentially expressed genes.** *bars indicate the likelihood [−log (*P*-value)] that the specific molecular and cellular function category was affected by the LP diet compared with the HP diet. The number of up- and down-regulated genes in each group is represented on the right hand side. The cut-off is shown at *P* < 0.05 (1.301 log scale).

**Figure 2:**
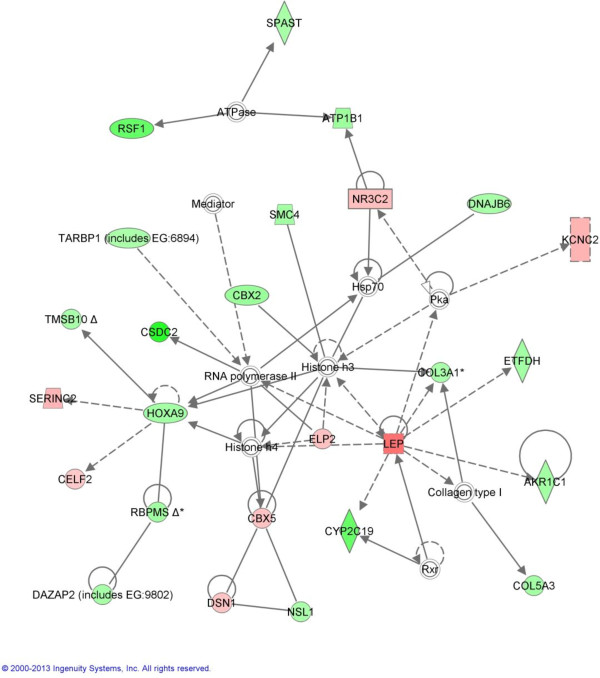
**Network 1: Molecular transport, lipid metabolism, small molecule biochemistry.** Molecules are represented as nodes, and the biological relationship between two nodes is represented as an edge (line). Up-regulated genes are indicated in red and down-regulated genes are indicated in green.

A total of 744 transcripts were differentially expressed (DE) at the 0.05 level (P-like values from puma). Of these, 542 genes were annotated by ANEXdb [22] and are presented in Additional file 1: Table S1. Fold changes ranged from −6.29 to 5.14 and 130 genes had a greater than 1.5 fold change across phenotypes. Previous studies of gene expression in relation to meat quality have shown that expression changes can be small [8,23] so in this study, all transcripts with a cut off *P*-like value of *P* ≤ 0.05 were considered DE. Differential expression assessed by realtime quantitative PCR (qPCR) for 13 selected genes was in a similar direction and magnitude compared to the microarray (Table 2). Although fold changes were generally low, a number of functional modules were altered and several broad patterns emerged through the various functional and ontological analyses. The most significant molecular and cellular functions, along with the number of up- and down-regulated genes observed in each function, are presented in Figure 1. The pathways significantly over-represented in the DE genes are presented in Table 3 and the most significant gene interaction network is presented in Figure 2, suggesting potential specific mechanisms underlying the broader biological functions. Taken together, these analyses suggest that an array of cellular processes including cell division and growth, protein metabolism, lipid metabolism, and the stress response were modulated in SM muscle by dietary protein restriction. Almost twice as many transcripts (351) were down-regulated, compared to up-regulated (191) indicating a repression of transcription in the SM muscle in response to protein restriction. Up- and down-regulated gene-lists were analysed separately using DAVID functional annotation clustering, permitting evaluation of the high-level trends among the DE genes, while reflecting the fact that genes have more than one function or may be members of several pathways. For up-regulated genes, three significant annotation clusters (AC) were identified which cluster terms relating to cell division, glucose metabolism and lipid metabolism, respectively (Table 4). Four AC were identified for the down-regulated gene-list (Table 5) with the first three of these all grouping terms related to cell division and cell cycle functions, while the fourth cluster represented terms related to extracellular matrix and connective tissue development. The major patterns observed in the overall dataset are discussed individually below.

**Table 2:** Genes selected for realtime quantitative PCR (qPCR) validation of differential expression observed on the microarray

**Gene category**	**Annotation probe ID**	**Gene symbol**	**Function**	**Microarray**		**qPCR**	
				FC*^#^	***P*-value**	FC*^#^	***P*-value**
Differentially expressed genes	Ssc.27307.1.S1_at	MPHOSPH6	Protein binding, RNA degradation	1.208	0.0009	1.413	0.006
	Ssc.24599.1.S1_at	UBE2CBP	Ligase activity	1.179	0.010	1.293	0.005
	Ssc.9340.1.A1_at	LTBR	Apoptosis	2.178	0.018	1.908	0.031
	Ssc.16159.1.S1_at	SCD	Synthesis of unsaturated fatty acids	2.374	0.011	6.2	0.066
	Ssc.17286.1.A1_at	BTG2	Protein binding	1.763	0.012	2.418	0.015
	Ssc.28320.1.S1_at	CBX5	Chromatin binding, enzyme binding	1.357	5.86E-07	1.214	0.091
	Ssc.17345.1.S1_at	ANGPTL4	Enzyme inhibitor activity, receptor binding	−2.204	7.3133E-06	−2.355	0.003
	Ssc.3020.1.A1_at	IQGAP2	Actin binding, cytoskeleton	−1.415	1.6622E-05	−1.358	0.098
	Ssc.26308.1.S1_at	PROCR-like	Receptor activity	−1.991	0.001	−2.461	0.013
	Ssc.27508.1.A1_at	SATB2	Protein coding, transcription factor	−1.409	7.1385E-05	−1.358	0.10
Non-changing genes	Ssc.11026.1.S1_at	RAP1	Cell adhesion and cell junction formation	1.000	0.98	1.267	0.16
	Ssc.14406.1.A1_at	MRPS6	Encodes ribosomal protein	1.001	0.96	1.241	0.22
	Ssc.1480.1.S1_at	PSMD1	Substrate recognition and binding	1.002	0.90	1.286	0.15

*FC: Fold change.

^#^Positive fold changes indicate that the low protein (LP) diet resulted in higher expression than the high protein (HP) diet and the number represents [expression LP/ expression HP]. Negative fold changes indicate that the LP diet resulted in lower expression than the HP diet and the number represents {−[expression HP / expression LP]}.

Gene expression changes (fold changes) and *P*-values determined by microarray and qPCR are shown.

**Table 3:** Gene classification according to canonical signalling pathways* using IPA

**Ingenuity® Canonical Pathways**	***P*-value**	**% DE**^#^	**Genes**
Factors Promoting Cardiogenesis in Vertebrates	0.0021	8.51	**TGFBR2**, **CCNE1**, TGFB2, **LEF1**, **TCF7L1**, APC, **PRKD1**, **CDK2**
Antiproliferative Role of TOB in T Cell Signalling	0.0047	15.4	**TGFBR2**, **CCNE1**,TGFB2, **CDK2**
Alanine and Aspartate Metabolism	0.0061	5.68	ADSL, **ASNSD1**, DARS, **MYO5B**, ASNS
HIF1α Signalling	0.0066	7.27	MAPK8, MRAS, KRAS, **MMP2**, HIF1A, MMP24, NOS2, P4HTM
Nicotinate and Nicotinamide Metabolism	0.018	5.15	NT5C3, DAPK1, ENPP1, NEK2, MAPK8, MAK, **CDK2**
Apoptosis Signalling	0.025	6.67	**CAPNS1**, MAPK8, MRAS, KRAS, **MAP4K4**, PARP1
PPARα/RXRα Activation	0.031	5	**TGFBR2**, GPD1, MAPK8, MRAS, TGFB2, KRAS, **MAP4K4**, **CYP2C19**, **ITGB5**
Arachidonic Acid Metabolism	0.037	3.57	**PLA2G4A**, **GPX3**, CYP4B1, **GPX5**, **ALOX12**, **CYP2C19**, **GPX6**, **PLOD3**
Nitrogen Metabolism	0.039	3.01	CA9, **CA3**, **ASNSD1**, ASNS
Prolactin Signalling	0.039	6.49	SOCS3, **SP1**, MRAS, KRAS, **PRKD1**
p38 MAPK Signalling	0.043	6.25	**TGFBR2**, **PLA2G4A**, DDIT3, TGFB2, **CREB3L4**, **HMGN1**
ATM Signalling	0.047	7.55	**CCNB1**, MAPK8, **CREB3L4**, **CDK2**
NF-κB Activation by Viruses	0.048	6.00	**CR2**, **ITGB5**, KRAS, MRAS, **PRKD1**
Intrinsic Prothrombin Activation Pathway	0.049	8.60	**COL3A1, COL5A3, PROS1**
HGF Signalling	0.049	5.71	MAPK8, MRAS, KRAS, **MAP3K3**, **PRKD1**, **CDK2**
TGF-β Signalling	0.054	6.00	KRAS, MAPK8, MRAS, TGFB2, **TGFBR2**

^#^% DE: Percentage of genes annotated in that pathway that were observed differentially expressed in the present study.

*Cancer, disease-specific pathways, neurotransmitter and other nervous system signalling and pathogen-influenced signalling were excluded from the analysis.

Down-regulated genes are highlighted in bold, up-regulated genes are in normal typeface.

**Table 4:** Functional annotation clustering of up-regulated genes by DAVID

**Annotation Cluster 1-**	**Count**	***P*-value**^**+**^	**Genes**
**Enrichment Score: 1.33**			
GO:0000087 ~ M phase of mitotic cell cycle	8	0.008	SSSCA1, FZR1, DDX11, NEK3, DSN1, RAN, MPHOSPH6, APC
GO:0000280 ~ nuclear division	7	0.025	SSSCA1, FZR1, DDX11, NEK3, DSN1, RAN, APC
GO:0007067 ~ mitosis	7	0.025	SSSCA1, FZR1, DDX11, NEK3, DSN1, RAN, APC
GO:0048285 ~ organelle fission	7	0.030	SSSCA1, FZR1, DDX11, NEK3, DSN1, RAN, APC
GO:0000278 ~ mitotic cell cycle	9	0.036	SSSCA1, FZR1, PSMA6, DDX11, NEK3, DSN1, RAN, MPHOSPH6, APC
**Annotation Cluster 2-**	**Count**	***P*-value**	**Genes**
**Enrichment Score: 1.26**			
GO:0010906 ~ regulation of glucose metabolic process	3	0.050	LEP, HIF1A, ECD
**Annotation Cluster 3-**	**Count**	***P*-value**	**Genes**
**Enrichment Score: 1.15**			
GO:0019637 ~ organophosphate metabolic process	7	0.017	SERINC2, GPD1, PIGU, PIGB, GALT, FIG 4, LIPC
GO:0046486 ~ glycerolipid metabolic process	6	0.025	SERINC2, GPD1, PIGU, PIGB, FIG 4, LIPC
GO:0006650 ~ glycerophospholipid metabolic process	5	0.033	SERINC2, PIGU, PIGB, FIG 4, LIPC

^+^*P*-value from modified Fisher exact score.

**Table 5:** Functional annotation clustering of down-regulated genes by DAVID

**Annotation Cluster 1-**	**Count**	***P*-value**^**+**^	**Genes**
**Enrichment Score: 2.63**			
GO:0051301 ~ cell division	18	3.317E-05	KIAA0892, NEK2, NUSAP1, CECR2, BIRC5, RACGAP1, MCM5, CDK2, SMC4, CCNB1, CCNE1, MAD2L1, NSL1, CKS2, BUB1, CHFR, ASPM, SPAST
GO:0000280 ~ nuclear division	13	0.0008	KIAA0892, NEK2, NUSAP1, AURKA, BIRC5, CDK2, SMC4, CCNB1, MAD2L1, NSL1, BUB1, CHFR, ASPM
GO:0007067 ~ mitosis	13	0.0008	KIAA0892, NEK2, NUSAP1, AURKA, BIRC5, CDK2, SMC4, CCNB1, MAD2L1, NSL1, BUB1, CHFR, ASPM
GO:0000087 ~ M phase of mitotic cell cycle	13	0.0009	KIAA0892, NEK2, NUSAP1, AURKA, BIRC5, CDK2, SMC4, CCNB1, MAD2L1, NSL1, BUB1, CHFR, ASPM
GO:0048285 ~ organelle fission	13	0.001	KIAA0892, NEK2, NUSAP1, AURKA, BIRC5, CDK2, SMC4, CCNB1, MAD2L1, NSL1, BUB1, CHFR, ASPM
GO:0000279 ~ M phase	16	0.001	KIAA0892, NEK2, NUSAP1, AURKA, BIRC5, PIM2, CDK2, SMC4, RPA1, CCNB1, MAD2L1, NSL1, CKS2, BUB1, CHFR, ASPM
GO:0007049 ~ cell cycle	27	0.002	CLSPN, NEK2, AURKA, SESN1, RPA1, CCNE1, BUB1, ASPM, KIAA0892, NUSAP1, BIRC5, PIM2, GAS1, RACGAP1, CDK2, SMC4, CCNB1, MAD2L1, NSL1, RASSF2, CKS2, G0S2, SIAH2, CHFR, CCNDBP1, SPAST, TP53INP1
GO:0007059 ~ chromosome segregation	7	0.004	KIAA0892, MAD2L1, NEK2, NSL1, NUSAP1, BIRC5, SMC4
GO:0022402 ~ cell cycle process	21	0.004	KIAA0892, NEK2, NUSAP1, AURKA, BIRC5, PIM2, GAS1, RACGAP1, SESN1, CDK2, SMC4, RPA1, CCNB1, CCNE1, MAD2L1, NSL1, CKS2, BUB1, CHFR, ASPM, TP53INP1
GO:0000070 ~ mitotic sister chromatid segregation	5	0.004	KIAA0892, MAD2L1, NEK2, NUSAP1, SMC4
GO:0022403 ~ cell cycle phase	17	0.004	KIAA0892, NEK2, NUSAP1, AURKA, BIRC5, PIM2, CDK2, SMC4, RPA1, CCNB1, CCNE1, MAD2L1, NSL1, CKS2, BUB1, CHFR, ASPM
GO:0000819 ~ sister chromatid segregation	5	0.005	KIAA0892, MAD2L1, NEK2, NUSAP1, SMC4
GO:0007346 ~ regulation of mitotic cell cycle	9	0.007	MAD2L1, HOXA13, NEK2, BUB1, NUSAP1, BIRC5, GAS1, CHFR, CDK2
GO:0000278 ~ mitotic cell cycle	14	0.020	KIAA0892, NEK2, NUSAP1, AURKA, BIRC5, CDK2, SMC4, CCNB1, CCNE1, MAD2L1, NSL1, BUB1, CHFR, ASPM
GO:0051726 ~ regulation of cell cycle	12	0.043	CCNB1, MAD2L1, HOXA13, TBX3, NEK2, BUB1, CKS2, NUSAP1, BIRC5, GAS1, CHFR, CDK2
**Annotation Cluster 2- Enrichment Score: 1.66**	**Count**	***P*-value**	**Genes**
GO:0007346 ~ regulation of mitotic cell cycle	9	0.007	MAD2L1, HOXA13, NEK2, BUB1, NUSAP1, BIRC5, GAS1, CHFR, CDK2
GO:0010564 ~ regulation of cell cycle process	7	0.019	MAD2L1, HOXA13, NEK2, BUB1, NUSAP1, BIRC5, GAS1
GO:0007088 ~ regulation of mitosis	5	0.019	MAD2L1, HOXA13, NEK2, BUB1, NUSAP1
GO:0051783 ~ regulation of nuclear division	5	0.019	MAD2L1, HOXA13, NEK2, BUB1, NUSAP1
GO:0051726 ~ regulation of cell cycle	12	0.043	CCNB1, MAD2L1, HOXA13, TBX3, NEK2, BUB1, CKS2, NUSAP1, BIRC5, GAS1, CHFR, CDK2
GO:0033043 ~ regulation of organelle organization	9	0.048	CAV1, PLA2G4A, MAD2L1, HOXA13, NEK2, ELN, BUB1, NUSAP1, TMSB10
**Annotation Cluster 3- Enrichment Score: 1.35**	**Count**	***P*-value**	**Genes**
GO:0010564 ~ regulation of cell cycle process	7	0.019	MAD2L1, HOXA13, NEK2, BUB1, NUSAP1, BIRC5, GAS1
GO:0031577 ~ spindle checkpoint	3	0.020	MAD2L1, BUB1, BIRC5
**Annotation Cluster 4- Enrichment Score: 1.33**	**Count**	***P*-value**	**Genes**
GO:0043062 ~ extracellular structure organization	9	0.011	GJA10, NRXN3, FBLN5, ELN, COL3A1, NID1, DCN, COL5A3, GFOD2
GO:0030198 ~ extracellular matrix organization	7	0.012	FBLN5, ELN, COL3A1, NID1, DCN, COL5A3, GFOD2
GO:0007160 ~ cell-matrix adhesion	6	0.024	LYVE1, FBLN5, COL3A1, ITGB5, NID1, COL5A3
GO:0031589 ~ cell-substrate adhesion	6	0.035	LYVE1, FBLN5, COL3A1, ITGB5, NID1, COL5A3

^+^*P*-value from modified Fisher exact score.

### Repression of cell cycle, cellular growth and tissue development

In response to the lack of available protein in the diet, animals on the LP diet appear to have had a reduced capacity for efficient growth (Table 1). Functional annotation of the DE genes highlighted a number of biological processes and those with the largest number of down-regulated genes were ‘*cellular growth and proliferation’*, ‘*tissue development’*, ‘*cell cycle’*, ‘*organismal development’* and ‘*skeletal and muscular system development’* (data from IPA). Of the observed genes which have functions in growth and development, greater numbers were down-regulated in tissue of animals on the LP diet than were up-regulated (Figure 1), suggesting these are the molecular mechanisms actively repressing growth in this model. They included groups of genes with important functions such as ‘*cellular assembly and organisation*’ (30 genes down-regulated; 12 up-regulated), ‘*cellular growth and proliferation*’ (79 down-regulated genes; 25 up-regulated) and ‘*cell cycle*’ (36 genes down-regulated; 20 up-regulated). IPA analysis (Table 3) revealed that key growth-related pathways modulated by the protein-restricted diet included, ‘*alanine and aspartate metabolism’,* ‘*TGF beta signalling*’, ‘*Arachidonic acid metabolism’* pathway ‘*nitrogen metabolism’*, and ‘*MAPK signalling*’, suggesting an overall repression of growth and development within the muscle tissue as a result of the LP diet, which is reflected in the reduced average daily gain and the tendency towards reduced carcass weight (Table 1). Functional annotation clustering of gene ontology term annotations in DAVID of down-regulated (n = 351) genes showed that many of the functional categories down-regulated in high IMF tissue relate to reduced availability of protein in the muscle, with significant categories such as ‘*skeletal system development’* and ‘*morphogenesis*’ indicating that these processes may be repressed in a restricted protein regime (Table 5). Three of the ACs related to cell division, cell cycle and reflected the divergence of resource allocation from protein deposition and growth. Despite the down-regulation of numerous protein synthesis and cellular growth pathways, there was no significant discrepancy in muscle depth or muscle protein concentration at slaughter between the two dietary conditions (Table 1).

Several of the observed pathways differentially expressed in muscle tissue, offer potential mechanisms, through which the restriction of dietary protein may act to mediate this inhibition of growth. The key difference between the two diets was the availability of dietary protein, which was energetically compensated for through inclusion of carbohydrate. A key indicator pathway of amino acid deprivation, ‘*alanine and aspartate metabolism’,* was significantly over-represented in the data (Table 3). For example, the asparagine synthetase gene, *ASNS* was significantly induced in LP diet muscle. *ASNS* responds to the deprivation of a wide range of different amino acids, therefore suggesting that this signal may be induced following amino acid restriction (Desvergne et al. 2006). This gene was also observed differentially expressed between lean and fatter pig breeds, i.e. Large White versus Basque [24]. A second significant pathway identified was the ‘*transforming growth factor-beta (TGF-β) signalling*’ pathway. The ‘*TGF-β signalling pathway’* was differentially expressed and *TGFBR* itself was down-regulated (Table 3). TGF-β plays key roles in regulating many cellular processes such as cell growth and differentiation, homeostasis, apoptosis, inflammation [25,26] and extracellular matrix (ECM) metabolism [27]. The ‘*Arachidonic acid metabolism’* pathway (Table 3) was also significantly modulated in the study. Arachidonic acid is involved in cellular signalling and is abundant in skeletal muscle [28]. Through its conversion to active components such as the prostaglandin PGF2α, arachidonic acid is necessary for the repair and growth of skeletal muscle tissue and in general muscle anabolic processes [29]. In the present study, the arachidonic acid pathway was also generally down-regulated in response to protein restriction (Table 3). Indeed, the second most repressed gene in the experiment with a −3.254 fold change was involved in arachidonic acid signalling i.e. potassium voltage-gated channel, Shal-related subfamily, member 2 (*KCND2*) [30]. Ramayo-Caldas et al. [31] also observed that the arachidonic acid pathway, including genes identified in the present study, such as *CYP2C19*, was significantly differentially expressed in liver of Iberian X Landrace pigs divergent for fatty acid content, suggesting this pathway may also be an influence on accumulation of fatty acids.

Since the protein and amino acid composition of the diet was extremely limiting (7 g/kg feed), in addition to the reduced availability of protein for growth, there may have been a reduced need for energy to excrete excess protein, as suggested by the down regulation of genes in the ‘*nitrogen metabolism’* pathway (Table 3). Furthermore, myofibrillar protein turnover may also have been restricted in high IMF tissue with observed down-regulation of the *CAPNS1* (Calpain small subunit 1) gene. The repression of protein turnover could contribute to the conservation and efficient utilisation of a reduced resource of dietary protein. The efficient response to dietary protein restriction may thus involve a suppression of both protein synthesis and breakdown pathways in order to achieve an overall improvement in the efficiency of protein accretion in the myofibrils.

### Extracellular matrix and connective tissue development

Connective tissue provides a source of physical strength and scaffolding support for growing muscle tissue [32,33]. In the present study, procollagen genes *COL3A1* (collagen, type III, alpha 1) and *COL5A3* (collagen type V, alpha 1) were prominent in the most significant network (Figure 2) and along with several other collagens (*COL8A1* and the only collagen strictly localised in the basement membrane *COL4A1*) were reduced in expression in high IMF tissue. Down-regulation of other genes important for collagen development e.g. *PLOD3* (pro-collagen-lysine, 2 oxoglutarate 5-dioxygenase 3), which codes for lysl hydroxylase and is involved in collagen synthesis [34] and *ALOX12* (arachidonate 12-lipoxygenase) was also seen in muscle of animals on the LP diet (Table 3). While the extracellular matrix was long considered a relatively inert ground substance, recent work has shown that it is an integral component of cellular signalling, through the interaction of extracellular matrix molecules with growth factors to influence cell shape, movement and gene expression [35]. For example, studies have shown that the removal of collagen VI stimulates the metabolism and survival of adipocytes [36]. Functional annotation clustering (Table 5) revealed that functions such as ‘*extracellular matrix organisation*’ and ‘*extracellular structure organisation*’ were represented in AC 4 amongst the down-regulated genes (e.g. *COL3A1, COL5A3, NID1, DCN*, *ITGB5* etc.). Damon and colleagues [24] investigated the *Longissimus* muscle transcriptome of two breeds contrasting for fatness, the highly selected Large White and the traditional unselected Basque breed, which displays lower production efficiency and higher IMF content compared to commercial breeds. They identified extracellular matrix functions as the most significantly over-represented group in their dataset. They also observed some of the genes identified in the present study such as decorin (*DCN*), which plays an important role in matrix assembly and whose impaired expression has a major influence on the collagen network. The extra-cellular matrix is an important influence underpinning differential cell and muscle tissue modelling. Identification of this component as being differentially expressed in two such contrasting animal models (dietary restriction and breed comparison) confirms its biological importance in the determination of composition and organisation of muscle tissue, as well as its significance to regulation of IMF.

### Modulation of lipid metabolism

Finisher pigs on the LP diet were characterised by a redirection of energy from overall growth to fat deposition, with increased fat accumulation in adipose depots including subcutaneous fat and IMF content (Table 1). This concept is supported by the observation that many of the up-regulated processes in SM muscle from animals on the LP diet have functions associated with increased lipid metabolism, and in particular synthesis (Tables 2, 3, 4 and Figures 1 and 2). IPA analysis of biological processes indicated that high IMF muscle displayed up-regulation of genes related to ‘*phospholipid metabolic process’, ‘glycerolipid biosynthetic process’, ‘lipid biosynthetic process’, ‘organophosphate metabolic process’,* as well as ‘*glucose metabolic process’, ‘regulation of cellular carbohydrate metabolic process’, ‘regulation of carbohydrate metabolic process’* and ‘*cell division’, ‘mitosis’, ‘mitotic cell cycle’.* Key pathways modulated by the protein-restricted diet include those involved with fatty acid uptake (‘*PPAR alpha/ RXR alpha*’) and fatty acid synthesis (‘*glycerolipid metabolic process*’ and ‘*glycerophospholipid metabolic process*’).

**Table 6:** Composition and chemical analysis of LP (12.8% protein) and HP (21.7% protein) diets

	**LP diet**	**HP diet**
**Ingredient (g/Kg)**		
Barley	200	200
Wheat	643	399.3
Soya bean meal	86	341
Tallow	42	34.2
Dical phosphate	10	6.2
Limestone	9.6	10.8
Salt	5	5
Lysine-HCL	2	1
Mineral and vitamins	2.5	2.5
Total	1000.0	1000
**Analysed Composition (g/Kg)**		
Dry Matter	885.0	890.0
Crude Protein (N x 6.25)	128.0	217.0
Ash	39.3	37.3
Crude Fibre	33.3	41.3
Crude Oil	25.1	34.3
Gross Energy (MJ)	15.7	15.8
Lysine	7.0	13.0
Methionine + Cysteine	4.2	7.8
Threonine	4.6	8.5
Tryptophan	1.3	2.3

Peroxisome proliferator activated receptor-alpha (PPARα) is a ligand-activated transcription factor that belongs to the nuclear receptor family. *PPARα* signalling plays major roles in the stimulation of uptake, binding, activation and consequent oxidation of fatty acids in several tissues including skeletal muscle [37]. *PPARα* is highly expressed in a number of tissues (skeletal and heart muscle, liver, and brown adipose tissue) with high levels of lipid catabolism and is activated by polyunsaturated fatty acids [38-40]. Here, differential expression of 9 genes involved in ‘*PPARα/RXRα activation’* (Table 1) was observed and these genes were in general up-regulated in the higher IMF condition. Canovas *et al.*[19] also observed induced *PPARα* signalling in muscle of pigs with high levels of IMF content and this is also reflected in liver of pigs divergent for fatty acid profile [31]. *PPARα* signalling was also highlighted in pathways relevant to carbohydrate metabolism indicating a high-level modulation of energy-generating and consuming pathways in muscle.

Network analysis and functional annotation of biological processes (Figure 2 and Table 4) revealed over-expression of a number of other genes with functions in lipid metabolism, including membrane lipid biosynthesis (e.g. *SERINC2, LTBR*, *MLYCD*, *PIGU, FIG 4, GPD1 etc.*), but which are not well known in relation to IMF accumulation. *SERINC2* (serine incorporator 2) is a member of the serine incorporator family which plays a pivotal role in the regulation of lipid biosynthesis by facilitating the synthesis of the serine-derived lipids, phosphatidylserine and sphingolipids [41]. Here, *SERINC2* was highly up-regulated and was a member of the top network. *SERINC2* has not been well-studied in the context of meat quality but was recently identified within a QTL for fatty acid profile [42]. Phospholipases are critical enzymes involved in mediating many aspects of cellular function through modulating the physical properties of membranes, generation of lipid second messengers and cellular bioenergetics. Cytosolic calcium-dependent phospholipase A2, group IVA (*PLA2G4A*) has been shown to mediate the increase in triglyceride content of adipose tissue after high-fat feeding [43] and here it was down-regulated in high IMF tissue. Lymphotoxin β Receptor (LTBR) is a member of the tumor necrosis factor receptor superfamily and has been shown to be an important mediator of lipid homeostasis [44]. Malonyl CoA decarboxylase (MLYCD) acts to increase the rate of fatty acid oxidation and *MLYCD* was down-regulated in the present study indicating that not only is lipid biosynthesis and accumulation up-regulated but that fatty acid turnover may be repressed. Stearoyl CoA Desaturase (SCD) is the catalyst of the conversion of saturated fatty acids to monounsaturated fatty acids (MUFA) [45,46] and is of interest from a health perspective and was highly up-regulated in the LP diet, confirming its association with IMF accumulation which is in accordance with observations by Canovas et. al [47] and da Costa et al. [15]. These genes are potential markers of fatty acid uptake, binding, storage and oxidation.

As well as its roles in cell growth and differentiation, expression of the *TGF-β* signalling pathway suppresses pre-adipoctye differentiation and reduces expression of glycerol-3-phosphate dehydrogenase (*GPD1*) [48]. The observed repression of the *TGF-β* signalling pathway and increased expression of *GPD1* in muscles of animals on the LP diet is thus a candidate mechanism for the increased orientation towards adipocyte differentiation and proliferation and may stimulate the accumulation of IMF in this model.

While the primary function of adipocytes are to store triacylglycerols during periods of energetic excess and release again during periods where energy is deprived, adipocytes are also a major source of endocrine and paracrine signals [1]. Adipogenic endocrine factors and secretory products are important mediators of energy homeostasis, immunological response, vascular disease and appetite regulation. ANGPTL4 (known as fasting-induced adipocyte factor), the hepatic fibrinogen/angiopoietin-related protein, or PPAR-γ angiopoietin-related protein, is a secreted glycoprotein abundantly expressed in adipocytes [49]. *ANGPTL4* abundance may be an indicator of IMF accumulation in porcine muscle. Network 1 (with functions in ‘*molecular transport’*, ‘*lipid metabolism’*, ‘*small molecule biochemistry’*) may be seen in Figure 2 and included 19 down-regulated and 7 up-regulated genes. Leptin (*LEP*) was a hub molecule in this network and was up-regulated and a number of its downstream genes were also altered (e.g. *ACSL1, MAP3K3, MAPK8*, *PNPLA3*). Leptin is secreted from adipose cells and acts as “lipostat” regulating food intake and energy expenditure to maintain body weight homeostasis [50,51]. Indeed, several studies have shown that high circulating levels of leptin inhibit fatty acid esterification in rodent [52] and porcine skeletal muscle [53] and promote lipid oxidation. Higher expression of *LEP* is expected when a greater number of adipocytes are present and when fat content of adipocytes is higher [49,54,55]. Our study supports the observed associations of *LEP* with high IMF accumulation in muscle tissue in line with previous findings [19].

### Induction of hypoxic stress response

Seven genes in the ‘*HIF1α signalling’* cascade were up-regulated (*P = 0.006*). Hypoxia-Inducible Factor (HIF1) is a transcription factor that regulates the homeostatic responses to hypoxia by inducing expression of proteins controlling glucose metabolism, cell proliferation, and angiogenesis [56]. The relatively larger expanse of adipose tissue in high IMF muscle, as is present in LP animals leads to the formation of hypoxic areas, as some adipocytes become too distant from the vasculature to receive oxygen properly [56,57]. *HIF1-α* expression has also been proposed elsewhere as a marker of IMF accumulation in pigs naturally divergent in IMF content [8]. Apoptosis in adipocytes can be induced through cell signalling in response to hypoxia, via the insulin-signalling pathway, leading to inhibition of glucose uptake and release of free fatty acids [58] and here the ‘*apoptosis signalling’* cascade was also significantly represented among the DE genes.

### Mechanisms regulating IMF accumulation in response to dietary protein restriction and other experimental models

Two previous studies have examined the influence of restriction of protein in porcine diet on gene expression and phenotype in porcine muscle. Da Costa et al. [15] examined energy and protein restriction and applied a small muscle specific microarray to male Duroc pigs on restricted and adequate energy and protein. Oster et al. [59] applied a reduced protein supply to the maternal diet during gestation; subsequently measuring phenotype and transcription in the offspring at different stages post mortem using the same array platform as employed in the present study. Da Costa et al. [15] in common with the present study, found reduced growth performance and increased IMF accumulation in pigs on restricted protein and energy diets. While in the present study, we have only studied one muscle, similarities were observed in the pathways and functions modulated across all three studies, and a number of pathways, e.g. fatty acid metabolism, were commonly differentially expressed in all three studies. Our phenotypic and gene expression findings in relation to dietary restriction at the post natal stage show interesting parallels with the impact of restricted protein at the gestational stage [59]. Restriction of growth performance was observed in the pigs restricted in protein during gestation as in those of the present study, which were restricted in protein in their own diets. Offspring offered poor protein supply during gestation had significantly lower body weight and lowered transcriptional abundance of growth-related genes and pathways at day 1 post natal. They compensated in terms of weight when offered adequate protein in later growth stages, but this was driven by fat accumulation while myofibrillar numbers remained low [59]. Despite reduced lean growth performance it was noteworthy however that major transcriptional differences as a result of protein restriction in gestation were not evident until approximately 188 days post natal. At this stage, pathways such as ‘*oxidative phosphorylation’*, ‘*fatty acid metabolism’*, ‘*cell cycle regulation’* and ‘*citrate cycle’* were differentially expressed in both model systems. This indicates that the major transcriptional shifts we have observed in the present study by restriction of protein in later stages of growth are also entailed by restriction of protein at the gestational stage. While dietary protein restriction is just one strategy to modify IMF content, a comparison of these studies and the study of Damon et al. [24], which applies a different model of differential IMF content, i.e. studying breeds contrasting for fatness, indicates that common metabolic processes may be induced by diverse stimuli such as genetics, nutrition and fetal programming. These common mechanisms lead to major differences in phenotypic outcomes for muscle tissue structure and organisation and give rise to important consequences for meat nutritional and eating quality.

## Conclusions

In this study, two extremes of IMF content were successfully generated using dietary lysine protein restriction. Remarkable differences were evident in the transcriptomic profiles. For example, LP muscle displayed over-expression of genes related to lipogenesis and lipolysis, while several genes known to be associated with adipoctye mass such as *SCD* and *LEP* were up-regulated. Functional categories which were decreased in their expression related to growth and development, protein synthesis and extracellular matrix development and organisation. The major patterns revealed by bioinformatic functional annotation clustering therefore involve restriction of cellular and muscle growth, cellular remodelling and differential expression of numerous lipid metabolic processes. A low protein diet can modulate IMF content by altering gene pathways involved in lipid biosynthesis and degradation; however this nutritional challenge negatively impacts protein synthesis pathways, which may have potential consequences for overall growth. The involvement of key master regulators of energy homeostasis illustrates the extensive perturbation of overall energy metabolism in muscle that occurs in response to protein restriction. Furthermore, the high IMF condition in this nutritional model is associated with a suite of responses which have also been observed in genetic and fetal programming models and thus may provide robust biological indicators of IMF accumulation.

## Methods

### Animals and treatments

All procedures described in this experiment were conducted under experimental licence from the Irish Department of Health in accordance with the Cruelty to Animals Act 1876 and the European Communities (Amendments of the cruelty of Animals Act 1976) Regulations, 1994. Eleven purebred Duroc gilts were blocked on the basis of initial body weight (BW) (46.3 ± 4.2 kg) and assigned to one of two dietary treatments. High (HP) and low protein (LP) diets were formulated to contain 217 and 128 g/kg crude protein (CP) respectively. Diet composition and nutrient analysis are detailed in Table 6. The HP and LP diets contained 13.0 and 7 g/kg of total lysine, respectively. Downward adjustment of CP was achieved by increasing the wheat:soyabean meal ratio. Diets were isocaloric, formulated to an estimated digestible energy (DE) density of 14 MJ/kg. Through the addition of synthetic amino acids, the dietary concentrations of threonine, tryptophan and total sulphur-containing amino acids were maintained at 65, 20 and 60% of lysine, respectively, which was sufficient to satisfy the ideal protein requirement [60]. The diets were manufactured into 3 mm pellets after heating the food to 85°C. Analysis of diets for dry matter (DM) and ash was carried out according to Association of Analytical Chemists (1995). Samples of feed were analysed for ether extract according to the Soxhlet method, using a Soxtec System (model 1043, Tecator, Sweden). The gross energy (GE) of feed and faeces samples was measured using an adiabatic bomb calorimeter (Parr Instruments, Il, USA). Dietary crude fibre was analysed by the Weende method [61] using a Fibertec system 1020 hot extractor (Tecator, Sweden). The dietary concentration of lysine, threonine, tryptophan and total sulphur amino acids was determined by high performance liquid chromatography [62]. The crude protein (CP, N x 6.25) concentration of feed was analysed by the macro-Kjeldahl technique using a Buchii distillation apparatus.

At approximately 10 weeks of age, six gilts were allocated to the HP diet and five gilts were allocated to the LP diet for 63 days. The pigs were penned in two groups with a space allowance of 0.75 m^2^ per pig. The house was mechanically ventilated to provide an ambient temperature of 18°C. Each pen had a solid floor lying area with access to slats. The pigs had *ad libitum* access to the diets. The pens were equipped with single-space computerized feeders (Mastleistungsprufung MLP-RAP; Schauer Agrotronic AG, Sursee, Switzerland) as described previously by Pauly et al. [63]. The pigs were weighed at the start of the experiment and subsequently on days 28 and 63 (morning of slaughter) and feed intake was recorded daily.

On day 63 of the experiment, animals were transported to the pilot-scale abattoir at Teagasc Food Research Centre, Ashtown. *M. semimembranosus* (SM) tissue was removed from each carcass, cut up finely under RNAse free conditions and preserved in RNALater® (Ambion) within 10 min post-slaughter, stored overnight at 4°C, and then stored at −20°C until RNA extraction. At day 1 post-mortem, SM muscle tissues were removed from the carcass, trimmed of subcutaneous fat and archived at −20°C for downstream compositional analysis.

#### Phenotype analysis

Fat and muscle depth were assessed on the carcass using the Hennessy Grading Probe according to supplier’s recommended protocol. Thawed muscle was cut into 3 cm^3^ cubes and homogenized using a Robot R301 Ultra Coupe blender (Robot Coupe Inc., MS, USA). IMF and moisture concentrations were determined using the Smart System 5 microwave moisture drying oven and NMR Smart Trac Rapid Fat Analyzer (CEM Corporation, Buckingham UK) using AOAC Official Methods 985.14 and 985.26, 1990. Protein concentration was determined using a LECO FP328 (LECO Corp., MI, USA) Protein Analyser based on the Dumas method and according to AOAC method 992.15, 1990. The least squares method of the GLM (General Linear Model) procedure in SAS [64] was carried out for statistical analysis of post-slaughter phenotypic and compositional measurements. Diet, slaughter weight and slaughter date were included in the model.

#### RNA extraction and cDNA synthesis

RNA extraction was carried out by Almac Diagnostics, UK. Briefly, preserved SM tissue samples were homogenised and processed using a standard phenol/guanidine isothiocyanate-based organic extraction method (e.g.TRI-Reagent, TRIZOL, or RNA STAT-60). RNA samples were analysed for concentration, purity and integrity using spectrophotometric and gel-based methods. Only samples with A260/280 ratios approximately 1.8-2.0 and two distinct peaks (for the 18S and 28S bands) were used to generate labelled targets.

For qPCR, one μg of DNAse-treated total RNA was used for cDNA synthesis using random primers and Superscript® III reverse transcriptase (Invitrogen Corp., San Diego, CA, USA). Negative control samples were prepared to ensure no genomic DNA contamination was present.

#### Microarray hybridisation

Gene expression profiling was performed using the Affymetrix GeneChip® Porcine Genome Array containing 23,937 probe sets that interrogate approximately 23,256 porcine transcripts from 20,201 *Sus scrofa* (pig) genes. RNA from each pig was hybridised to a separate array. Hybridisation, processing and preliminary analysis of porcine SM muscle RNA samples were carried out by Almac Diagnostics, UK, according to the manufacturer’s instructions. Briefly, 100 ng of total RNA was amplified using the NuGEN™ Ovation™ RNA Amplification System V2. After obtaining the first double-stranded cDNA, the appropriate amount of amplified single-stranded cDNA was used as a template for biotin labelling using the FL-Ovation™ cDNA Biotin Module V2. The enzymatically and chemically fragmented product (50–100 bp) was then labelled via the attachment of biotinylated nucleotides onto the 3′-end of the fragmented cDNA. The resultant fragmented and labelled cDNA was added to the hybridisation mix in accordance with the NuGEN™ guidelines for hybridisation onto Affymetrix GeneChip® arrays. Post hybridisation washing and staining were carried out using GeneChip® Fluidics Station 450 (Affymetrix) followed by scanning according to standard Affymetrix protocols.

#### Data analysis and bioinformatics

Microarray analysis including, pre-processing, normalisation and statistical analysis were performed using R (R, 2007) version 2.6 and Bioconductor [65] version 2.1., as previously described by Morris et al. [66]. Quality of data was determined before and after normalisation using a number of in-built QC methods as implemented in the Bioconductor Affycoretools and integrated packages to visualise problems if any existed, following array hybridisation, RNA degradation and data normalisation. The Propagating Uncertainty in Microarray Analysis (puma) method was used to estimate fold changes and P-like values for differential gene expression [67-69]. The puma package permits the use of probe level uncertainty to give improved performance when compared to traditional methods of differential gene expression analysis, principal component analysis and clustering. It uses a Bayesian hierarchical model to calculate the probability of positive likelihood ratio (PPLR) of differential gene expression. The PPLR associates probability values of genes being differentially expressed, which is a measure of the false positive detection of differential expression, to each ratio and generates lists of genes ranked by the probability of being DE. This PPLR statistic was converted into P-like values using the recommended formula in the puma method prior to subsequent analysis. The data set, which is compliant to the MIAME criteria, is deposited at Gene Expression Omnibus (GEO, http://www.ncbi.nlm.nih.gov/geo/) under the series accession number GSE44326.

#### Annotation and ontology analysis

ANEXdb (an integrated animal ANnotation and microarray EXpression database) [22] was used for annotation of DE genes. Following annotation, gene symbols were submitted to DAVID (Database for Annotation, Visualization and Integrated Discovery [70,71] with the purpose of determining gene function and to cluster genes into functionally significant gene clusters and also to determine significant biological pathways through KEGG (Kyoto Encyclopaedia of Genes and Genomes: [72,73]. Ingenuity Pathway Analysis (IPA, version 8.7, Ingenuity® Systems, CA, USA) was used for functional classification of DE genes. IPA is web-based software that allows identification of pathways, biological network and functions of experimental data and gene lists. In order to identify pathways that were more relevant to the metabolic processes of interest, cancer, disease-specific, neurotransmitter, nervous system signalling and pathogen-influenced signalling pathways were excluded from the IPA analysis.

#### Realtime quantitative PCR (qPCR) validation of selected DE genes

Gene specific primers were designed online using the Primer3 software (http://frodo.wi.mit.edu/primer3/) based on the porcine target sequences used on the Affymetrix array. All corresponding consensus expressed sequences were derived using the NetAffx Analysis Centre (http://www.affymetrix.com/analysis/index.affx) on the website. Specificity of primers was determined using the BLAST search tool on NCBI database (http://www.ncbi.nlm.nih.gov/BLAST). Oligonucleotides were commercially synthesised (Eurofins, Germany) and an initial conventional PCR reaction confirmed for each target gene that all RNA samples were free of contaminating genomic DNA with one distinct band present on a 1.2% agarose gel.

Primer sequences for three selected reference genes *Beta 2 microglobulin (β2M), TATA box binding protein* (*TBP)* and *Ribosomal protein L4 (RPL4*) are described in detail in McBryan et al. [74]. qPCR was performed on 96-well plates and reactions were carried out in triplicate for each cDNA sample. One μl of cDNA was amplified in a 20 μl volume using 10 μl SYBR® Green master mix and 1 μl of 0.2 μM forward and reverse primer mix and 8 μl nuclease free water (QIAGEN Ltd., West Sussex, UK). Cycling conditions for reference and selected genes on the 7500 system (Applied Biosystems, Foster City, CA, USA) were as follows: 50°C for 2 min, 95°C for 10 min, 40 cycles of 95°C for 15 s and 60°C for 1 min, 95°C for 15 s, 60°C for 1 min, 95°C for 15 s. All reactions included a dissociation curve analysis at the end of the amplification to confirm a single product at the expected melting temperature. Relative expression values were determined by comparison to a standard curve using serial dilutions of a pool of cDNA. The relative stability of the three reference genes was analysed using GeNorm [75].

Ten DE genes (six up-regulated: *MPHOSPH6, UBE2CBP, PNPLA3, SCD, BTG2, CBX5* and four down-regulated: *ANGPTL4, IQGAP2, PROCR, SATB2*) were selected based on their fold changes and their known functional roles in lipid, amino acid or skeletal muscle metabolism. In addition to these genes, three non-changing genes (*RAP1, MRPS6* and *PSMD1*) were also selected for the confirmation of microarray analysis. The thirteen genes (ten DE genes and three non-changing genes) with details of accession number, gene symbol, primer sequences, and product size are listed in Additional file 1: Table S2.

## Competing interests

The authors declare that they have no competing interests.

## Authors’ contributions

RMH carried out animal sampling, meat quality analysis, participated in experimental design, gene expression analysis, data analysis and interpretation, drafting and editing of the manuscript. OA participated in animal sampling, composition analysis and qPCR and participated in drafting of the manuscript. AMM participated in experimental design, data interpretation and editing of the manuscript. JOD carried out the animal feeding trial and participated in experimental design and editing of the manuscript. JMB participated in qPCR analysis. DGM carried out bioinformatic analysis of microarray data. TS participated in experimental design, data interpretation and editing of the manuscript. All authors read and approved the final manuscript.

## Supplementary Material

Additional file 1: Table S1 542 differentially expressed probesets and associated annotations from Anexdb. **Table S2**. Primer details for reference, differentially expressed and non-changing genes. Click here for file
