# Supplementary material for: Transcriptome analysis of porcine M. semimembranosus divergent in intramuscular fat as a consequence of dietary protein restriction
Source: BMC Genomics. 2013 Jul 6;14:453. doi: 10.1186/1471-2164-14-453 (PMC3710489; doi:10.1186/1471-2164-14-453)
Supplement: Additional file 1: Table S1 — 542 differentially expressed probesets and associated annotations from Anexdb. Table S2. Primer details for reference, differentially expressed and non-changing genes. [file 1471-2164-14-453-S1.doc]

**Supplementary Table 1 - 542 annotated differentially expressed probesets (*P < 0.05*) and associated annotations from Anexdb (**[**http://www.anexdb.org**](http://www.anexdb.org/)**)**

| **Affymetrix Probe ID** | **Fold Change^#^** | ***P* - like value** | **Gene Symbol** | **RefSeq ID** | **RefSeq Description** |
| --- | --- | --- | --- | --- | --- |
| Ssc.5344.2.S1_at | 5.14 | 0.0002 | ANKS1B | NM_181670.2 | Homo sapiens ankyrin repeat and sterile alpha motif domain containing 1B (ANKS1B); transcript variant 2; mRNA |
| Ssc.18226.1.S1_at | 4.86 | 0.0416 | AURKAIP1 | NM_001127230.1 | Homo sapiens aurora kinase A interacting protein 1 (AURKAIP1); transcript variant 3; mRNA |
| Ssc.2827.1.S1_at | 4.59 | 0.0122 | SOCS3 | NM_003955.3 | Homo sapiens suppressor of cytokine signaling 3 (SOCS3); mRNA |
| Ssc.265.1.S3_at | 3.20 | 0.0002 | LEP | NM_000230.2 | Homo sapiens leptin (LEP); mRNA |
| Ssc.27433.1.S1_at | 3.13 | 0.0009 | TGM1 | NM_000359.2 | Homo sapiens transglutaminase 1 (K polypeptide epidermal type I; protein-glutamine-gamma-glutamyltransferase) (TGM1); mRNA |
| Ssc.18239.2.S1_at | 2.90 | 0.0026 | DIS3L2 | NM_152383.4 | Homo sapiens DIS3 mitotic control homolog (S. cerevisiae)-like 2 (DIS3L2); mRNA |
| Ssc.11370.1.A1_at | 2.45 | 0.0080 | LMBR1 | NM_022458.3 | Homo sapiens limb region 1 homolog (mouse) (LMBR1); mRNA |
| Ssc.16159.1.S1_at | 2.37 | 0.0108 | SCD | NM_005063.4 | Homo sapiens stearoyl-CoA desaturase (delta-9-desaturase) (SCD); mRNA |
| Ssc.16105.1.S1_at | 2.28 | 0.0358 | BHMT | NM_001713.2 | Homo sapiens betaine-homocysteine methyltransferase (BHMT); mRNA |
| Ssc.11031.1.A1_at | 2.25 | 0.0003 | SLC11A2 | NM_000617.1 | Homo sapiens solute carrier family 11 (proton-coupled divalent metal ion transporters); member 2 (SLC11A2); mRNA >gi|2911111|dbj|AB004857.1| Homo sapiens mRNA for NRAMP2; complete cds |
| Ssc.25880.1.A1_at | 2.24 | 0.0322 | hCG_2008140 | NR_024279.1 | Homo sapiens hypothetical LOC729614 (FLJ37453); non-coding RNA |
| Ssc.9340.1.A1_at | 2.18 | 0.0180 | LTBR | NM_002342.1 | Homo sapiens lymphotoxin beta receptor (TNFR superfamily; member 3) (LTBR); mRNA >gi|339761|gb|L04270.1|HUMTNFRRP Homo sapiens (clone CD18) tumor necrosis factor receptor 2 related protein mRNA; complete cds |
| Ssc.15791.1.S1_at | 2.12 | 0.0036 | IL12RB2 | NM_001559.2 | Homo sapiens interleukin 12 receptor; beta 2 (IL12RB2); mRNA |
| Ssc.16526.2.S1_at | 2.07 | 0.0239 | ARHGEF16 | NM_014448.3 | Homo sapiens Rho guanine exchange factor (GEF) 16 (ARHGEF16); mRNA |
| Ssc.19980.1.S1_at | 2.01 | 0.0400 | ADAM28 | NM_014265.4 | Homo sapiens ADAM metallopeptidase domain 28 (ADAM28); transcript variant 1; mRNA |
| Ssc.16117.1.A1_at | 2.01 | 0.0109 | NOS2 | NM_000625.4 | Homo sapiens nitric oxide synthase 2; inducible (NOS2); mRNA |
| Ssc.19211.1.S1_at | 1.96 | 0.0013 | CYP4B1 | NM_000779.3 | Homo sapiens cytochrome P450; family 4; subfamily B; polypeptide 1 (CYP4B1); transcript variant 2; mRNA |
| Ssc.15694.1.S1_at | 1.92 | 0.0359 | MT3 | NM_005954.2 | Homo sapiens metallothionein 3 (MT3); mRNA |
| Ssc.12183.1.A1_at | 1.91 | 0.0273 | GRIA1 | NM_001114183.1 | Homo sapiens glutamate receptor; ionotropic; AMPA 1 (GRIA1); transcript variant 2; mRNA |
| Ssc.28259.1.A1_at | 1.86 | 0.0228 | SLC25A25 | NM_001006642.1 | Homo sapiens solute carrier family 25 (mitochondrial carrier; phosphate carrier); member 25 (SLC25A25); nuclear gene encoding mitochondrial protein; transcript variant 3; mRNA |
| Ssc.18278.3.A1_at | 1.86 | 0.0002 | HABP2 | NM_004132.3 | Homo sapiens hyaluronan binding protein 2 (HABP2); mRNA |
| Ssc.20663.1.S1_at | 1.85 | 0.0130 | MOBP | NR_003090.1 | Homo sapiens myelin-associated oligodendrocyte basic protein (MOBP); transcribed RNA |
| Ssc.30264.1.A1_at | 1.85 | 0.0328 | MTMR7 | NM_004686.3 | Homo sapiens myotubularin related protein 7 (MTMR7); mRNA |
| Ssc.8086.1.A1_at | 1.84 | 0.0091 | LMO3 | NM_001001395.1 | Homo sapiens LIM domain only 3 (rhombotin-like 2) (LMO3); transcript variant 2; mRNA |
| Ssc.27578.1.S1_at | 1.83 | 0.0014 | MAK | NM_005906.3 | Homo sapiens male germ cell-associated kinase (MAK); mRNA |
| Ssc.6276.2.S1_at | 1.82 | 0.0028 | ABHD6 | NM_020676.5 | Homo sapiens abhydrolase domain containing 6 (ABHD6); mRNA |
| Ssc.25030.1.A1_at | 1.81 | 0.0499 | SEMA6A | NM_020796.3 | Homo sapiens sema domain; transmembrane domain (TM); and cytoplasmic domain; (semaphorin) 6A (SEMA6A); mRNA |
| Ssc.8484.1.A1_at | 1.80 | 0.0405 | OSBPL6 | NM_145739.1 | Homo sapiens oxysterol binding protein-like 6 (OSBPL6); transcript variant 2; mRNA |
| Ssc.28995.3.S1_at | 1.79 | 0.0037 | ELMOD3 | NM_001135023.1 | Homo sapiens ELMO/CED-12 domain containing 3 (ELMOD3); transcript variant 4; mRNA |
| Ssc.25444.1.S1_at | 1.77 | 0.0102 | MAGEB3 | NM_002365.3 | Homo sapiens melanoma antigen family B; 3 (MAGEB3); mRNA |
| Ssc.30902.1.A1_at | 1.77 | 0.0172 | PKHD1L1 | NM_177531.4 | Homo sapiens polycystic kidney and hepatic disease 1 (autosomal recessive)-like 1 (PKHD1L1); mRNA |
| Ssc.9707.1.A1_at | 1.76 | 0.0126 | BTG2 | NM_006763.2 | Homo sapiens BTG family; member 2 (BTG2); mRNA |
| Ssc.26443.1.S1_at | 1.73 | 0.0233 | NUDT16 | NM_152395.1 | Homo sapiens nudix (nucleoside diphosphate linked moiety X)-type motif 16 (NUDT16); mRNA >gi|16550653|dbj|AK055827.1| Homo sapiens cDNA FLJ31265 fis; clone KIDNE2006030; moderately similar to Gallus gallus syndesmos mRNA |
| Ssc.26022.1.S1_at | 1.72 | 0.0322 | LOC728951 | XM_001128885.2 | PREDICTED: Homo sapiens hypothetical LOC728951 (LOC728951); mRNA |
| Ssc.24298.1.S1_at | 1.72 | 0.0160 | TMEFF1 | NM_003692.3 | Homo sapiens transmembrane protein with EGF-like and two follistatin-like domains 1 (TMEFF1); mRNA |
| Ssc.24321.1.A1_at | 1.71 | 0.0251 | GRIA2 | NM_001083620.1 | Homo sapiens glutamate receptor; ionotropic; AMPA 2 (GRIA2); transcript variant 3; mRNA |
| Ssc.1139.1.A1_at | 1.68 | 0.0154 | SLC4A4 | NM_001134742.1 | Homo sapiens solute carrier family 4; sodium bicarbonate cotransporter; member 4 (SLC4A4); transcript variant 3; mRNA |
| Ssc.18180.2.S1_at | 1.68 | 0.0242 | KIAA0319L | NM_024874.3 | Homo sapiens KIAA0319-like (KIAA0319L); transcript variant 1; mRNA |
| Ssc.13524.1.A1_at | 1.67 | 0.0176 | KCNC2 | NM_153748.1 | Homo sapiens potassium voltage-gated channel; Shaw-related subfamily; member 2 (KCNC2); transcript variant 3; mRNA |
| Ssc.2999.2.S1_at | 1.65 | 0.0040 | SERINC2 | NM_178865.3 | Homo sapiens serine incorporator 2 (SERINC2); mRNA |
| Ssc.11899.1.A1_at | 1.65 | 0.0314 | NLE1 | NM_018096.3 | Homo sapiens notchless homolog 1 (Drosophila) (NLE1); transcript variant 1; mRNA |
| Ssc.24188.1.A1_at | 1.61 | 0.0406 | PF4 | NM_002619.2 | Homo sapiens platelet factor 4 (PF4); mRNA |
| Ssc.162.1.S1_at | 1.60 | 0.0187 | RLN2 | NM_005059.2 | Homo sapiens relaxin 2 (RLN2); transcript variant 2; mRNA |
| Ssc.27532.1.A1_at | 1.59 | 0.0000 | ATL2 | NM_022374.2 | Homo sapiens atlastin GTPase 2 (ATL2); transcript variant 1; mRNA |
| Ssc.15142.1.A1_at | 1.59 | 0.0422 | WWP2 | NM_199424.1 | Homo sapiens WW domain containing E3 ubiquitin protein ligase 2 (WWP2); transcript variant 2; mRNA |
| Ssc.16038.1.S1_at | 1.59 | 0.0170 | HLA-DOA | NM_002119.3 | Homo sapiens major histocompatibility complex; class II; DO alpha (HLA-DOA); mRNA |
| Ssc.30710.1.S1_at | 1.58 | 0.0173 | IPO9 | NM_018085.4 | Homo sapiens importin 9 (IPO9); mRNA |
| Ssc.3455.3.A1_at | 1.56 | 0.0005 | UBAP2L | NM_001127320.1 | Homo sapiens ubiquitin associated protein 2-like (UBAP2L); transcript variant 2; mRNA |
| Ssc.24086.1.A1_at | 1.56 | 0.0182 | ENAH | NM_018212.4 | Homo sapiens enabled homolog (Drosophila) (ENAH); transcript variant 2; mRNA |
| Ssc.21692.1.A1_at | 1.54 | 0.0067 | C14orf102 | NM_199043.1 | Homo sapiens chromosome 14 open reading frame 102 (C14orf102); transcript variant 2; mRNA |
| Ssc.21999.1.S1_a_at | 1.52 | 0.0469 | ALDH3B1 | NM_000694.2 | Homo sapiens aldehyde dehydrogenase 3 family; member B1 (ALDH3B1); transcript variant 1; mRNA |
| Ssc.25358.1.S1_at | 1.51 | 0.0497 | GRAMD2 | NM_001012642.1 | Homo sapiens GRAM domain containing 2 (GRAMD2); mRNA >gi|21754262|dbj|AK095072.1| Homo sapiens cDNA FLJ37753 fis; clone BRHIP2023438; weakly similar to GRAM domain-containing protein 3 |
| Ssc.27140.1.A1_at | 1.50 | 0.0027 | PTPN2 | NM_002828.2 | Homo sapiens protein tyrosine phosphatase; non-receptor type 2 (PTPN2); transcript variant 1; mRNA |
| Ssc.16126.1.A1_at | 1.49 | 0.0477 | ATP1A2 | NM_000702.3 | Homo sapiens ATPase; Na+/K+ transporting; alpha 2 (+) polypeptide (ATP1A2); mRNA |
| Ssc.18303.3.S1_at | 1.48 | 0.0468 | ZNF385D | NM_024697.1 | Homo sapiens zinc finger protein 385D (ZNF385D); mRNA >gi|10438803|dbj|AK026072.1| Homo sapiens cDNA: FLJ22419 fis; clone HRC08593 |
| Ssc.16605.1.S1_at | 1.47 | 0.0003 | DDIT3 | NM_004083.4 | Homo sapiens DNA-damage-inducible transcript 3 (DDIT3); mRNA |
| Ssc.11425.1.A1_at | 1.47 | 0.0026 | SLC35B4 | NM_032826.4 | Homo sapiens solute carrier family 35; member B4 (SLC35B4); mRNA |
| Ssc.18377.1.S1_at | 1.46 | 0.0327 | P2RX4 | NM_002560.2 | Homo sapiens purinergic receptor P2X; ligand-gated ion channel; 4 (P2RX4); mRNA |
| Ssc.11149.2.S1_at | 1.46 | 0.0111 | CA9 | NM_001216.2 | Homo sapiens carbonic anhydrase IX (CA9); mRNA |
| Ssc.19105.1.S1_at | 1.44 | 0.0467 | LIPC | NM_000236.2 | Homo sapiens lipase; hepatic (LIPC); mRNA |
| Ssc.22500.1.S1_at | 1.44 | 0.0000 | BPGM | NM_199186.1 | Homo sapiens 2;3-bisphosphoglycerate mutase (BPGM); transcript variant 2; mRNA |
| Ssc.17877.1.A1_at | 1.43 | 0.0002 | NR3C2 | NM_000901.3 | Homo sapiens nuclear receptor subfamily 3; group C; member 2 (NR3C2); mRNA |
| Ssc.19239.1.S1_at | 1.41 | 0.0371 | GPD1 | NM_005276.2 | Homo sapiens glycerol-3-phosphate dehydrogenase 1 (soluble) (GPD1); mRNA >gi|21594876|gb|BC032234.1| Homo sapiens glycerol-3-phosphate dehydrogenase 1 (soluble); mRNA (cDNA clone MGC:34464 IMAGE:5229925); complete cds |
| Ssc.17286.1.A1_at | 1.40 | 0.0467 | BTG2 | NM_006763.2 | Homo sapiens BTG family; member 2 (BTG2); mRNA |
| Ssc.25048.1.A1_at | 1.40 | 0.0177 | P4HTM | NM_177939.2 | Homo sapiens hypoxia-inducible factor prolyl 4-hydroxylase (PH-4); transcript variant 1; mRNA |
| Ssc.3577.1.S1_at | 1.39 | 0.0247 | DDX11 | NM_030653.3 | Homo sapiens DEAD/H (Asp-Glu-Ala-Asp/His) box polypeptide 11 (CHL1-like helicase homolog; S. cerevisiae) (DDX11); transcript variant 1; mRNA |
| Ssc.24232.1.S1_at | 1.39 | 0.0002 | ELP2 | NM_018255.1 | Homo sapiens elongation protein 2 homolog (S. cerevisiae) (ELP2); mRNA >gi|7023192|dbj|AK001741.1| Homo sapiens cDNA FLJ10879 fis; clone NT2RP4001896; weakly similar to VEGETATIBLE INCOMPATIBILITY PROTEIN HET-E-1 |
| Ssc.27413.1.S1_at | 1.37 | 0.0290 | ECD | NR_024203.1 | Homo sapiens ecdysoneless homolog (Drosophila) (ECD); transcript variant 4; transcribed RNA |
| Ssc.11281.1.A1_at | 1.37 | 0.0071 | PENK | NM_001135690.1 | Homo sapiens proenkephalin (PENK); transcript variant 1; mRNA |
| Ssc.121.1.S1_at | 1.36 | 0.0161 | PDYN | NM_024411.2 | Homo sapiens prodynorphin (PDYN); mRNA |
| Ssc.28320.1.S1_at | 1.36 | 0.0000 | CBX5 | NM_012117.2 | Homo sapiens chromobox homolog 5 (HP1 alpha homolog; Drosophila) (CBX5); transcript variant 3; mRNA |
| Ssc.11609.1.A1_at | 1.36 | 0.0141 | ASNS | NM_183356.2 | Homo sapiens asparagine synthetase (ASNS); transcript variant 3; mRNA |
| Ssc.825.1.S1_at | 1.36 | 0.0137 | CLDN7 | NM_001307.4 | Homo sapiens claudin 7 (CLDN7); mRNA |
| Ssc.9208.1.A1_at | 1.35 | 0.0179 | ANKRD10 | NM_017664.2 | Homo sapiens ankyrin repeat domain 10 (ANKRD10); mRNA |
| Ssc.25248.2.S1_at | 1.35 | 0.0308 | MED9 | NM_018019.2 | Homo sapiens mediator complex subunit 9 (MED9); mRNA |
| Ssc.22176.1.A1_at | 1.35 | 0.0022 | APC | NM_001127511.1 | Homo sapiens adenomatous polyposis coli (APC); transcript variant 1; mRNA |
| Ssc.14125.1.A1_at | 1.34 | 0.0256 | NEK3 | NM_002498.2 | Homo sapiens NIMA (never in mitosis gene a)-related kinase 3 (NEK3); transcript variant 1; mRNA |
| Ssc.7064.1.A1_at | 1.34 | 0.0212 | CCDC38 | NM_182496.1 | Homo sapiens coiled-coil domain containing 38 (CCDC38); mRNA >gi|21757152|dbj|AK097408.1| Homo sapiens cDNA FLJ40089 fis; clone TESTI2003181 |
| Ssc.23945.1.A1_at | 1.34 | 0.0144 | TET1 | NM_030625.2 | Homo sapiens tet oncogene 1 (TET1); mRNA |
| Ssc.29136.1.S1_at | 1.34 | 0.0127 | GPATCH4 | NM_182679.1 | Homo sapiens G patch domain containing 4 (GPATCH4); transcript variant 2; mRNA |
| Ssc.10287.1.A1_at | 1.33 | 0.0088 | TGFB2 | NM_003238.2 | Homo sapiens transforming growth factor; beta 2 (TGFB2); transcript variant 2; mRNA |
| Ssc.2472.1.S1_at | 1.32 | 0.0295 | CYB5D2 | NM_144611.3 | Homo sapiens cytochrome b5 domain containing 2 (CYB5D2); transcript variant 1; mRNA |
| Ssc.29036.1.S1_at | 1.32 | 0.0115 | TUBA4A | NM_006000.1 | Homo sapiens tubulin; alpha 4a (TUBA4A); mRNA |
| Ssc.4320.1.S1_at | 1.32 | 0.0035 | IKZF4 | NM_022465.3 | Homo sapiens IKAROS family zinc finger 4 (Eos) (IKZF4); mRNA |
| Ssc.13891.1.A1_at | 1.31 | 0.0309 | RGN | NM_152869.2 | Homo sapiens regucalcin (senescence marker protein-30) (RGN); transcript variant 2; mRNA |
| Ssc.18642.1.S1_at | 1.31 | 0.0325 | ITGB1BP2 | NM_012278.1 | Homo sapiens integrin beta 1 binding protein (melusin) 2 (ITGB1BP2); mRNA >gi|6017903|gb|AF140690.1|AF140690 Homo sapiens melusin mRNA; complete cds |
| Ssc.16814.2.S1_at | 1.31 | 0.0087 | C19orf61 | NM_019108.2 | Homo sapiens chromosome 19 open reading frame 61 (C19orf61); mRNA |
| Ssc.2291.2.S1_at | 1.30 | 0.0003 | GYS1 | NM_002103.3 | Homo sapiens glycogen synthase 1 (muscle) (GYS1); mRNA >gi|33988671|gb|BC002617.2| Homo sapiens glycogen synthase 1 (muscle); mRNA (cDNA clone MGC:2986 IMAGE:3143019); complete cds |
| Ssc.17567.2.S1_at | 1.30 | 0.0288 | MMP24 | NM_006690.3 | Homo sapiens matrix metallopeptidase 24 (membrane-inserted) (MMP24); mRNA |
| Ssc.2250.1.A1_at | 1.30 | 0.0001 | FBXW7 | NM_018315.4 | Homo sapiens F-box and WD repeat domain containing 7 (FBXW7); transcript variant 2; mRNA |
| Ssc.29222.2.S1_at | 1.30 | 0.0106 | UBE2CBP | NM_198920.1 | Homo sapiens ubiquitin-conjugating enzyme E2C binding protein (UBE2CBP); mRNA >gi|38347756|dbj|AB126062.1| Homo sapiens H10BH mRNA for UbcH10 binding protein with a hect-like domain; complete cds |
| Ssc.23768.1.S1_at | 1.30 | 0.0335 | RTF1 | NM_015138.4 | Homo sapiens Rtf1; Paf1/RNA polymerase II complex component; homolog (S. cerevisiae) (RTF1); mRNA |
| Ssc.21327.1.S1_at | 1.29 | 0.0031 | ST3GAL3 | NM_006279.2 | Homo sapiens ST3 beta-galactoside alpha-2;3-sialyltransferase 3 (ST3GAL3); transcript variant 10; mRNA |
| Ssc.11488.3.A1_at | 1.29 | 0.0230 | DSN1 | NM_024918.2 | Homo sapiens DSN1; MIND kinetochore complex component; homolog (S. cerevisiae) (DSN1); mRNA >gi|21751761|dbj|AK093031.1| Homo sapiens cDNA FLJ35712 fis; clone TESOP1000160 |
| Ssc.26753.1.A1_at | 1.29 | 0.0128 | CACNA2D1 | NM_000722.2 | Homo sapiens calcium channel; voltage-dependent; alpha 2/delta subunit 1 (CACNA2D1); mRNA |
| Ssc.2679.1.S1_at | 1.29 | 0.0001 | SMYD5 | NM_006062.2 | Homo sapiens SMYD family member 5 (SMYD5); mRNA |
| Ssc.24214.1.S1_at | 1.28 | 0.0000 | GRWD1 | NM_031485.2 | Homo sapiens glutamate-rich WD repeat containing 1 (GRWD1); mRNA >gi|22760271|dbj|AK074676.1| Homo sapiens cDNA FLJ90195 fis; clone MAMMA1001310 |
| Ssc.30644.1.S1_at | 1.28 | 0.0166 | PKN3 | NM_013355.3 | Homo sapiens protein kinase N3 (PKN3); mRNA |
| Ssc.11364.1.S1_at | 1.28 | 0.0133 | FIG4 | NM_014845.5 | Homo sapiens FIG4 homolog (S. cerevisiae) (FIG4); mRNA |
| Ssc.4733.1.A1_at | 1.27 | 0.0208 | CHIC1 | NM_001039840.2 | Homo sapiens cysteine-rich hydrophobic domain 1 (CHIC1); mRNA |
| Ssc.4087.1.A1_at | 1.27 | 0.0003 | C4orf16 | NM_001128426.1 | Homo sapiens chromosome 4 open reading frame 16 (C4orf16); transcript variant 2; mRNA |
| Ssc.2105.1.A1_at | 1.27 | 0.0138 | AKAP5 | NM_004857.3 | Homo sapiens A kinase (PRKA) anchor protein 5 (AKAP5); mRNA |
| Ssc.31184.1.S1_at | 1.27 | 0.0043 | YBX2 | NM_015982.3 | Homo sapiens Y box binding protein 2 (YBX2); mRNA |
| Ssc.390.1.A1_at | 1.27 | 0.0244 | HIF1A | NM_181054.2 | Homo sapiens hypoxia-inducible factor 1; alpha subunit (basic helix-loop-helix transcription factor) (HIF1A); transcript variant 2; mRNA |
| Ssc.23809.3.S1_at | 1.26 | 0.0008 | TNRC6B | NM_015088.2 | Homo sapiens trinucleotide repeat containing 6B (TNRC6B); transcript variant 1; mRNA |
| Ssc.8371.1.A1_at | 1.26 | 0.0009 | RAN | NM_006325.2 | Homo sapiens RAN; member RAS oncogene family (RAN); mRNA |
| Ssc.9382.1.A1_at | 1.26 | 0.0361 | PSMA6 | NM_002791.1 | Homo sapiens proteasome (prosome; macropain) subunit; alpha type; 6 (PSMA6); mRNA |
| Ssc.9548.1.A1_at | 1.25 | 0.0003 | COG5 | NM_006348.2 | Homo sapiens component of oligomeric golgi complex 5 (COG5); transcript variant 1; mRNA |
| Ssc.92.1.S1_at | 1.25 | 0.0322 | TGFB2 | NM_003238.2 | Homo sapiens transforming growth factor; beta 2 (TGFB2); transcript variant 2; mRNA |
| Ssc.1918.1.S1_at | 1.25 | 0.0117 | MRPS7 | NM_015971.3 | Homo sapiens mitochondrial ribosomal protein S7 (MRPS7); nuclear gene encoding mitochondrial protein; mRNA |
| Ssc.4265.1.A1_a_at | 1.25 | 0.0247 | TNRC6B | NM_015088.2 | Homo sapiens trinucleotide repeat containing 6B (TNRC6B); transcript variant 1; mRNA |
| Ssc.26457.1.A1_at | 1.25 | 0.0015 | TIGD1 | NM_145702.1 | Homo sapiens tigger transposable element derived 1 (TIGD1); mRNA >gi|16551701|dbj|AK056329.1| Homo sapiens cDNA FLJ31767 fis; clone NT2RI2007884; weakly similar to ARS BINDING PROTEIN 1 |
| Ssc.29671.1.A1_at | 1.25 | 0.0468 | INTS8 | NM_017864.2 | Homo sapiens integrator complex subunit 8 (INTS8); mRNA >gi|42407297|dbj|AB161944.1| Homo sapiens mRNA for KAONASHI protein 1; complete cds >gi|78100166|tpg|BK005731.1| TPA_exp: Homo sapiens integrator complex subunit 8 mRNA; complete cds |
| Ssc.7444.2.S1_at | 1.25 | 0.0261 | CREM | NM_001881.2 | Homo sapiens cAMP responsive element modulator (CREM); transcript variant 2; mRNA |
| Ssc.17264.1.S1_at | 1.25 | 0.0329 | SLC25A1 | NM_005984.2 | Homo sapiens solute carrier family 25 (mitochondrial carrier; citrate transporter); member 1 (SLC25A1); nuclear gene encoding mitochondrial protein; mRNA |
| Ssc.1405.1.S1_at | 1.24 | 0.0030 | PIGU | NM_080476.4 | Homo sapiens phosphatidylinositol glycan anchor biosynthesis; class U (PIGU); mRNA >gi|38564692|gb|AY422169.1| Homo sapiens transamidase complex subunit PIG-U (PIGU) mRNA; complete cds |
| Ssc.18243.2.S1_at | 1.23 | 0.0459 | BTBD9 | NM_001099272.1 | Homo sapiens BTB (POZ) domain containing 9 (BTBD9); transcript variant 2; mRNA |
| Ssc.828.1.S1_at | 1.23 | 0.0113 | SLC26A6 | NM_134426.2 | Homo sapiens solute carrier family 26; member 6 (SLC26A6); transcript variant 3; mRNA |
| Ssc.23860.1.A1_at | 1.23 | 0.0004 | MAPK8 | NM_002750.2 | Homo sapiens mitogen-activated protein kinase 8 (MAPK8); transcript variant JNK1-a1; mRNA |
| Ssc.1388.1.S1_at | 1.23 | 0.0023 | NUDT2 | NM_001161.3 | Homo sapiens nudix (nucleoside diphosphate linked moiety X)-type motif 2 (NUDT2); transcript variant 1; mRNA |
| Ssc.2250.2.S1_at | 1.23 | 0.0225 | FBXW7 | NM_033632.2 | Homo sapiens F-box and WD repeat domain containing 7 (FBXW7); transcript variant 1; mRNA |
| Ssc.21616.1.S1_at | 1.23 | 0.0211 | ARHGAP10 | NM_024605.3 | Homo sapiens Rho GTPase activating protein 10 (ARHGAP10); mRNA |
| Ssc.23809.1.S1_at | 1.23 | 0.0215 | TNRC6B | NM_015088.2 | Homo sapiens trinucleotide repeat containing 6B (TNRC6B); transcript variant 1; mRNA |
| Ssc.7239.1.S1_a_at | 1.22 | 0.0044 | ADSL | NM_001123378.1 | Homo sapiens adenylosuccinate lyase (ADSL); transcript variant 2; mRNA |
| Ssc.16954.2.S1_at | 1.22 | 0.0431 | TFPT | NM_013342.2 | Homo sapiens TCF3 (E2A) fusion partner (in childhood Leukemia) (TFPT); mRNA |
| Ssc.16111.2.S1_at | 1.22 | 0.0379 | PMVK | NM_006556.3 | Homo sapiens phosphomevalonate kinase (PMVK); mRNA |
| Ssc.9630.1.A1_at | 1.22 | 0.0373 | CNKSR1 | NR_023345.1 | Homo sapiens connector enhancer of kinase suppressor of Ras 1 (CNKSR1); transcript variant 2; transcribed RNA |
| Ssc.11730.1.S1_at | 1.21 | 0.0004 | SCAF1 | NM_021228.1 | Homo sapiens SR-related CTD-associated factor 1 (SCAF1); mRNA >gi|32450501|gb|BC053992.1| Homo sapiens SR-related CTD-associated factor 1; mRNA (cDNA clone MGC:45499 IMAGE:5578439); complete cds |
| Ssc.12819.1.A1_at | 1.21 | 0.0424 | MON1B | NM_014940.2 | Homo sapiens MON1 homolog B (yeast) (MON1B); mRNA |
| Ssc.27307.1.S1_at | 1.21 | 0.0009 | MPHOSPH6 | NM_005792.2 | Homo sapiens M-phase phosphoprotein 6 (MPHOSPH6); mRNA |
| Ssc.18243.3.S1_a_at | 1.21 | 0.0093 | BTBD9 | NM_152733.2 | Homo sapiens BTB (POZ) domain containing 9 (BTBD9); transcript variant 3; mRNA |
| Ssc.18574.1.A1_at | 1.20 | 0.0154 | CUGBP2 | NM_001083591.1 | Homo sapiens CUG triplet repeat; RNA binding protein 2 (CUGBP2); transcript variant 4; mRNA |
| Ssc.18185.1.S1_at | 1.20 | 0.0096 | HYPK | NM_016400.2 | Homo sapiens Huntingtin interacting protein K (HYPK); mRNA >gi|17939528|gb|BC019262.1| Homo sapiens Huntingtin interacting protein K; mRNA (cDNA clone MGC:2632 IMAGE:3504222); complete cds |
| Ssc.11417.1.A1_at | 1.20 | 0.0366 | FAM126A | NM_032581.3 | Homo sapiens family with sequence similarity 126; member A (FAM126A); mRNA |
| Ssc.24930.1.S1_at | 1.20 | 0.0182 | LTV1 | NM_032860.3 | Homo sapiens LTV1 homolog (S. cerevisiae) (LTV1); mRNA >gi|33872922|gb|BC009855.2| Homo sapiens LTV1 homolog (S. cerevisiae); mRNA (cDNA clone MGC:16354 IMAGE:3926227); complete cds |
| Ssc.22328.3.S1_at | 1.20 | 0.0307 | DARS | NM_001349.2 | Homo sapiens aspartyl-tRNA synthetase (DARS); mRNA |
| Ssc.24483.1.A1_at | 1.19 | 0.0011 | PRPF40A | NM_017892.3 | Homo sapiens PRP40 pre-mRNA processing factor 40 homolog A (S. cerevisiae) (PRPF40A); mRNA |
| Ssc.10936.2.A1_at | 1.19 | 0.0079 | KIAA0232 | NM_001100590.1 | Homo sapiens KIAA0232 (KIAA0232); transcript variant 2; mRNA |
| Ssc.28600.1.A1_at | 1.19 | 0.0256 | MFSD8 | NM_152778.2 | Homo sapiens major facilitator superfamily domain containing 8 (MFSD8); mRNA |
| Ssc.5520.1.S1_at | 1.19 | 0.0420 | SEPX1 | NM_016332.2 | Homo sapiens selenoprotein X; 1 (SEPX1); mRNA |
| Ssc.2439.1.S1_at | 1.19 | 0.0061 | WDR13 | NM_017883.3 | Homo sapiens WD repeat domain 13 (WDR13); mRNA |
| Ssc.2392.1.A1_at | 1.19 | 0.0367 | CCNL1 | NM_020307.2 | Homo sapiens cyclin L1 (CCNL1); mRNA |
| Ssc.19964.1.A1_a_at | 1.18 | 0.0038 | NFU1 | NM_001002755.1 | Homo sapiens NFU1 iron-sulfur cluster scaffold homolog (S. cerevisiae) (NFU1); transcript variant 2; mRNA |
| Ssc.24795.1.A1_at | 1.18 | 0.0364 | WDR76 | NM_024908.2 | Homo sapiens WD repeat domain 76 (WDR76); mRNA |
| Ssc.15255.1.S1_at | 1.18 | 0.0249 | CATSPER2 | NM_172095.1 | Homo sapiens cation channel; sperm associated 2 (CATSPER2); transcript variant 2; mRNA |
| Ssc.23265.1.A1_at | 1.18 | 0.0304 | RNF160 | NM_015565.1 | Homo sapiens zinc finger protein 294 (ZNF294); mRNA |
| Ssc.24599.1.S1_at | 1.18 | 0.0107 | UBE2CBP | NM_198920.1 | Homo sapiens ubiquitin-conjugating enzyme E2C binding protein (UBE2CBP); mRNA >gi|38347756|dbj|AB126062.1| Homo sapiens H10BH mRNA for UbcH10 binding protein with a hect-like domain; complete cds |
| Ssc.18581.1.S1_at | 1.18 | 0.0076 | USP5 | NM_003481.2 | Homo sapiens ubiquitin specific peptidase 5 (isopeptidase T) (USP5); transcript variant 2; mRNA |
| Ssc.18422.1.S1_a_at | 1.18 | 0.0346 | DNASE1L1 | NM_006730.2 | Homo sapiens deoxyribonuclease I-like 1 (DNASE1L1); transcript variant 1; mRNA |
| Ssc.29092.1.A1_at | 1.18 | 0.0490 | KRAS | NM_004985.3 | Homo sapiens v-Ki-ras2 Kirsten rat sarcoma viral oncogene homolog (KRAS); transcript variant b; mRNA |
| Ssc.1639.1.A1_at | 1.18 | 0.0222 | GALT | NM_000155.2 | Homo sapiens galactose-1-phosphate uridylyltransferase (GALT); mRNA |
| Ssc.1363.1.S1_at | 1.17 | 0.0218 | FDX1L | NM_001031734.2 | Homo sapiens ferredoxin 1-like (FDX1L); mRNA |
| Ssc.2729.1.S1_at | 1.17 | 0.0329 | BNIP1 | NM_013980.2 | Homo sapiens BCL2/adenovirus E1B 19kDa interacting protein 1 (BNIP1); transcript variant BNIP1-c; mRNA |
| Ssc.30475.1.A1_at | 1.17 | 0.0003 | PIGB | NM_004855.4 | Homo sapiens phosphatidylinositol glycan anchor biosynthesis; class B (PIGB); mRNA |
| Ssc.16686.1.A1_at | 1.17 | 0.0135 | FAM48A | NM_001014286.2 | Homo sapiens family with sequence similarity 48; member A (FAM48A); transcript variant 1; mRNA |
| Ssc.18640.3.S1_at | 1.17 | 0.0181 | UBE2D4 | NM_015983.2 | Homo sapiens ubiquitin-conjugating enzyme E2D 4 (putative) (UBE2D4); mRNA >gi|7022709|dbj|AK001446.1| Homo sapiens cDNA FLJ10584 fis; clone NT2RP2003737; highly similar to UBIQUITIN-CONJUGATING ENZYME E2-17 KD 2 (EC 6.3.2.19) |
| Ssc.15894.1.S1_at | 1.17 | 0.0454 | FZR1 | NM_016263.3 | Homo sapiens fizzy/cell division cycle 20 related 1 (Drosophila) (FZR1); transcript variant 2; mRNA |
| Ssc.20489.1.S1_at | 1.17 | 0.0480 | MDK | NM_002391.3 | Homo sapiens midkine (neurite growth-promoting factor 2) (MDK); transcript variant 3; mRNA |
| Ssc.26703.1.S1_at | 1.16 | 0.0412 | C16orf87 | NM_001001436.2 | Homo sapiens chromosome 16 open reading frame 87 (C16orf87); mRNA |
| Ssc.6112.1.S1_at | 1.16 | 0.0170 | UBR1 | NM_174916.2 | Homo sapiens ubiquitin protein ligase E3 component n-recognin 1 (UBR1); mRNA |
| Ssc.8506.1.A1_at | 1.16 | 0.0118 | RBM25 | NM_021239.1 | Homo sapiens RNA binding motif protein 25 (RBM25); mRNA >gi|34366144|emb|BX647116.1|HSM807260 Homo sapiens mRNA; cDNA DKFZp686M06144 (from clone DKFZp686M06144) |
| Ssc.1481.1.S1_at | 1.16 | 0.0024 | JARID1A | NM_001042603.1 | Homo sapiens jumonji; AT rich interactive domain 1A (JARID1A); transcript variant 1; mRNA |
| Ssc.9300.1.A1_at | 1.16 | 0.0477 | REV1 | NM_016316.2 | Homo sapiens REV1 homolog (S. cerevisiae) (REV1); transcript variant 1; mRNA |
| Ssc.9634.1.A1_at | 1.16 | 0.0295 | RANBP6 | NR_024095.1 | Homo sapiens RAN binding protein 6 (RANBP6); transcript variant 3; transcribed RNA |
| Ssc.12256.1.S1_at | 1.16 | 0.0240 | CCDC104 | NM_080667.5 | Homo sapiens coiled-coil domain containing 104 (CCDC104); mRNA |
| Ssc.23006.1.S1_at | 1.16 | 0.0492 | NUCKS1 | NM_022731.3 | Homo sapiens nuclear casein kinase and cyclin-dependent kinase substrate 1 (NUCKS1); mRNA |
| Ssc.27060.1.A1_at | 1.15 | 0.0428 | SSSCA1 | NM_006396.1 | Homo sapiens Sjogren syndrome/scleroderma autoantigen 1 (SSSCA1); mRNA >gi|2982672|dbj|AB001740.1| Homo sapiens mRNA for p27; complete cds |
| Ssc.25124.1.S1_at | 1.15 | 0.0237 | EPC2 | NM_015630.3 | Homo sapiens enhancer of polycomb homolog 2 (Drosophila) (EPC2); mRNA |
| Ssc.27513.1.A1_at | 1.15 | 0.0341 | PARP1 | NM_001618.3 | Homo sapiens poly (ADP-ribose) polymerase 1 (PARP1); mRNA |
| Ssc.26167.1.S1_at | 1.15 | 0.0146 | ST3GAL3 | NM_174963.1 | Homo sapiens ST3 beta-galactoside alpha-2;3-sialyltransferase 3 (ST3GAL3); transcript variant 1; mRNA |
| Ssc.15421.2.S1_at | 1.15 | 0.0077 | C20orf43 | NM_016407.3 | Homo sapiens chromosome 20 open reading frame 43 (C20orf43); mRNA |
| Ssc.6249.2.S1_at | 1.15 | 0.0205 | OCIAD1 | NM_017830.2 | Homo sapiens OCIA domain containing 1 (OCIAD1); transcript variant 1; mRNA |
| Ssc.7699.1.A1_at | 1.14 | 0.0160 | ADAT2 | NM_182503.2 | Homo sapiens adenosine deaminase; tRNA-specific 2; TAD2 homolog (S. cerevisiae) (ADAT2); mRNA |
| Ssc.16496.1.S1_at | 1.14 | 0.0411 | WDR76 | NM_024908.2 | Homo sapiens WD repeat domain 76 (WDR76); mRNA |
| Ssc.21947.1.S1_at | 1.14 | 0.0133 | MAP1D | NM_199227.1 | Homo sapiens methionine aminopeptidase 1D (MAP1D); mRNA >gi|38893020|gb|AY374142.1| Homo sapiens mitochondrial methionine aminopeptidase 1 mRNA; complete cds; nuclear gene for mitochondrial product |
| Ssc.5212.1.S1_at | 1.14 | 0.0293 | UIMC1 | NM_016290.3 | Homo sapiens ubiquitin interaction motif containing 1 (UIMC1); mRNA >gi|31873253|emb|BX537376.1|HSM805671 Homo sapiens mRNA; cDNA DKFZp686C17196 (from clone DKFZp686C17196) |
| Ssc.22152.1.A1_at | 1.14 | 0.0255 | USE1 | NM_018467.3 | Homo sapiens unconventional SNARE in the ER 1 homolog (S. cerevisiae) (USE1); mRNA |
| Ssc.20347.1.S1_at | 1.14 | 0.0479 | TMEM194B | XM_001726342.1 | PREDICTED: Homo sapiens similar to hCG1646803 (LOC100131211); mRNA |
| Ssc.23524.1.S1_at | 1.13 | 0.0264 | EXOSC10 | NM_001001998.1 | Homo sapiens exosome component 10 (EXOSC10); transcript variant 1; mRNA |
| Ssc.12010.1.A1_at | 1.13 | 0.0304 | ZRSR2 | NM_005089.3 | Homo sapiens zinc finger (CCCH type); RNA-binding motif and serine/arginine rich 2 (ZRSR2); mRNA |
| Ssc.27119.1.A1_at | 1.13 | 0.0334 | TTC4 | NM_004623.3 | Homo sapiens tetratricopeptide repeat domain 4 (TTC4); mRNA |
| Ssc.17756.1.S1_at | 1.13 | 0.0157 | MRAS | NM_012219.3 | Homo sapiens muscle RAS oncogene homolog (MRAS); transcript variant 1; mRNA |
| Ssc.2786.1.S1_at | 1.12 | 0.0466 | ITGA7 | NM_002206.1 | Homo sapiens integrin; alpha 7 (ITGA7); mRNA >gi|2897115|gb|AF032108.1|AF032108 Homo sapiens integrin alpha-7 mRNA; complete cds |
| Ssc.11627.1.A1_at | 1.12 | 0.0408 | RBM23 | NM_001077352.1 | Homo sapiens RNA binding motif protein 23 (RBM23); transcript variant 3; mRNA |
| Ssc.7351.1.S1_at | 1.12 | 0.0358 | BTBD3 | NM_181443.1 | Homo sapiens BTB (POZ) domain containing 3 (BTBD3); transcript variant 2; mRNA |
| Ssc.9139.1.A1_at | 1.11 | 0.0354 | TRDMT1 | NM_004412.4 | Homo sapiens tRNA aspartic acid methyltransferase 1 (TRDMT1); transcript variant a; mRNA |
| Ssc.1709.1.S1_at | 1.11 | 0.0264 | LOC727773 | XM_001126273.2 | PREDICTED: Homo sapiens similar to p28 ING5; transcript variant 2 (LOC727773); mRNA |
| Ssc.10598.1.A1_at | 1.11 | 0.0424 | ZFYVE16 | NM_001105251.1 | Homo sapiens zinc finger; FYVE domain containing 16 (ZFYVE16); transcript variant 2; mRNA |
| Ssc.10082.1.A1_at | 1.11 | 0.0405 | GNPTAB | NM_024312.3 | Homo sapiens N-acetylglucosamine-1-phosphate transferase; alpha and beta subunits (GNPTAB); mRNA |
| Ssc.13759.2.S1_at | 1.11 | 0.0353 | TUBGCP2 | NM_006659.2 | Homo sapiens tubulin; gamma complex associated protein 2 (TUBGCP2); mRNA |
| Ssc.16529.1.S1_at | 1.10 | 0.0208 | SNX6 | NM_152233.2 | Homo sapiens sorting nexin 6 (SNX6); transcript variant 2; mRNA |
| Ssc.7062.1.A1_at | -1.10 | 0.0468 | ASNSD1 | NM_019048.1 | Homo sapiens asparagine synthetase domain containing 1 (ASNSD1); mRNA >gi|7021046|dbj|AK000759.1| Homo sapiens cDNA FLJ20752 fis; clone HEP02921 |
| Ssc.20951.1.S1_at | -1.10 | 0.0423 | METTL5 | NM_014168.2 | Homo sapiens methyltransferase like 5 (METTL5); mRNA >gi|12803007|gb|BC000921.2| Homo sapiens methyltransferase like 5; mRNA (cDNA clone MGC:4937 IMAGE:3445582); complete cds |
| Ssc.12285.1.A1_at | -1.10 | 0.0483 | AMMECR1L | NM_031445.2 | Homo sapiens AMME chromosomal region gene 1-like (AMMECR1L); mRNA |
| Ssc.19029.1.A1_at | -1.11 | 0.0430 | WAPAL | NM_015045.2 | Homo sapiens wings apart-like homolog (Drosophila) (WAPAL); mRNA |
| Ssc.2835.1.S1_at | -1.11 | 0.0467 | HNRNPUL1 | NM_144732.2 | Homo sapiens heterogeneous nuclear ribonucleoprotein U-like 1 (HNRNPUL1); transcript variant 4; mRNA |
| Ssc.9188.1.A1_at | -1.11 | 0.0441 | NSL1 | NM_001042549.1 | Homo sapiens NSL1; MIND kinetochore complex component; homolog (S. cerevisiae) (NSL1); transcript variant 2; mRNA |
| Ssc.1131.2.A1_at | -1.11 | 0.0311 | SLC9A6 | NM_006359.2 | Homo sapiens solute carrier family 9 (sodium/hydrogen exchanger); member 6 (SLC9A6); transcript variant 2; mRNA |
| Ssc.11053.1.S1_at | -1.11 | 0.0451 | SRP19 | NM_003135.1 | Homo sapiens signal recognition particle 19kDa (SRP19); mRNA >gi|36112|emb|X12791.1|HSRP19 Human mRNA for 19kD protein of signal recognition particle (SRP) |
| Ssc.5265.1.S1_at | -1.11 | 0.0418 | CHFR | NM_018223.1 | Homo sapiens checkpoint with forkhead and ring finger domains (CHFR); mRNA >gi|7023050|dbj|AK001658.1| Homo sapiens cDNA FLJ10796 fis; clone NT2RP4000648; weakly similar to TRANS-ACTING TRANSCRIPTIONAL PROTEIN ICP0 |
| Ssc.4357.1.S1_at | -1.11 | 0.0139 | PTRF | NM_012232.3 | Homo sapiens polymerase I and transcript release factor (PTRF); mRNA |
| Ssc.3061.1.A1_at | -1.11 | 0.0499 | CRLS1 | NM_001127458.1 | Homo sapiens cardiolipin synthase 1 (CRLS1); transcript variant 2; mRNA |
| Ssc.11278.2.A1_at | -1.11 | 0.0379 | ZNF2 | NM_001017396.1 | Homo sapiens zinc finger protein 2 (ZNF2); transcript variant 2; mRNA |
| Ssc.21684.1.S1_at | -1.11 | 0.0240 | PHF16 | NM_001077445.1 | Homo sapiens PHD finger protein 16 (PHF16); transcript variant 2; mRNA |
| Ssc.18848.1.S1_at | -1.12 | 0.0492 | TARBP1 | NM_005646.3 | Homo sapiens TAR (HIV-1) RNA binding protein 1 (TARBP1); mRNA |
| Ssc.865.2.A1_at | -1.12 | 0.0173 | LASS2 | NM_181746.2 | Homo sapiens LAG1 homolog; ceramide synthase 2 (LASS2); transcript variant 1; mRNA |
| Ssc.11114.1.A1_at | -1.12 | 0.0432 | RPA1 | NM_002945.3 | Homo sapiens replication protein A1; 70kDa (RPA1); mRNA |
| Ssc.19229.1.S1_at | -1.12 | 0.0276 | RABGAP1L | NM_014857.3 | Homo sapiens RAB GTPase activating protein 1-like (RABGAP1L); transcript variant 1; mRNA |
| Ssc.30480.1.A1_at | -1.13 | 0.0473 | TBL1XR1 | NM_024665.4 | Homo sapiens transducin (beta)-like 1 X-linked receptor 1 (TBL1XR1); mRNA |
| Ssc.20134.1.A1_at | -1.13 | 0.0013 | UBE2J1 | NM_016021.2 | Homo sapiens ubiquitin-conjugating enzyme E2; J1 (UBC6 homolog; yeast) (UBE2J1); mRNA |
| Ssc.11302.1.S1_at | -1.13 | 0.0395 | COL3A1 | NM_000090.3 | Homo sapiens collagen; type III; alpha 1 (COL3A1); mRNA |
| Ssc.3255.1.S1_at | -1.13 | 0.0171 | ZFYVE21 | NM_024071.2 | Homo sapiens zinc finger; FYVE domain containing 21 (ZFYVE21); mRNA >gi|13543697|gb|BC005999.1| Homo sapiens zinc finger; FYVE domain containing 21; mRNA (cDNA clone MGC:14803 IMAGE:4091809); complete cds |
| Ssc.2569.1.S1_at | -1.13 | 0.0377 | TSPAN14 | NM_001128309.1 | Homo sapiens tetraspanin 14 (TSPAN14); transcript variant 2; mRNA |
| Ssc.6053.1.A1_at | -1.13 | 0.0378 | USP46 | NM_001134223.1 | Homo sapiens ubiquitin specific peptidase 46 (USP46); transcript variant 2; mRNA |
| Ssc.11302.1.S2_at | -1.13 | 0.0488 | COL3A1 | NM_000090.3 | Homo sapiens collagen; type III; alpha 1 (COL3A1); mRNA |
| Ssc.6559.1.S1_at | -1.13 | 0.0381 | RPA2 | NM_002946.3 | Homo sapiens replication protein A2; 32kDa (RPA2); mRNA >gi|33878123|gb|BC021257.2| Homo sapiens replication protein A2; 32kDa; mRNA (cDNA clone MGC:29683 IMAGE:4111194); complete cds |
| Ssc.9170.1.A1_at | -1.13 | 0.0420 | PRKD1 | NM_002742.2 | Homo sapiens protein kinase D1 (PRKD1); mRNA |
| Ssc.7067.1.A1_at | -1.13 | 0.0427 | TSPAN6 | NM_003270.2 | Homo sapiens tetraspanin 6 (TSPAN6); mRNA |
| Ssc.1527.2.A1_at | -1.13 | 0.0441 | SLC20A1 | NM_005415.3 | Homo sapiens solute carrier family 20 (phosphate transporter); member 1 (SLC20A1); mRNA >gi|18044776|gb|BC019944.1| Homo sapiens solute carrier family 20 (phosphate transporter); member 1; mRNA (cDNA clone MGC:8767 IMAGE:3918690); complete cds |
| Ssc.2240.1.S1_at | -1.14 | 0.0493 | TMEM55B | NM_001100814.1 | Homo sapiens transmembrane protein 55B (TMEM55B); transcript variant 1; mRNA |
| Ssc.21845.2.S1_at | -1.14 | 0.0128 | GTPBP10 | NM_033107.2 | Homo sapiens GTP-binding protein 10 (putative) (GTPBP10); transcript variant 2; mRNA |
| Ssc.15730.1.S1_at | -1.14 | 0.0242 | RAMP2 | NM_005854.2 | Homo sapiens receptor (G protein-coupled) activity modifying protein 2 (RAMP2); mRNA |
| Ssc.17230.1.A1_at | -1.14 | 0.0189 | TMSB10 | NM_021103.3 | Homo sapiens thymosin beta 10 (TMSB10); mRNA |
| Ssc.26249.1.S1_at | -1.14 | 0.0414 | CCNDBP1 | NM_012142.2 | Homo sapiens cyclin D-type binding-protein 1 (CCNDBP1); transcript variant 1; mRNA |
| Ssc.14342.1.A1_at | -1.14 | 0.0117 | MSL1 | NM_001012241.1 | Homo sapiens male-specific lethal 1 homolog (Drosophila) (MSL1); mRNA |
| Ssc.5330.1.A1_at | -1.14 | 0.0405 | TGFBR2 | NM_003242.5 | Homo sapiens transforming growth factor; beta receptor II (70/80kDa) (TGFBR2); transcript variant 2; mRNA |
| Ssc.4091.1.S1_at | -1.14 | 0.0130 | KIAA0892 | NM_015329.3 | Homo sapiens KIAA0892 (KIAA0892); mRNA |
| Ssc.4584.1.S1_at | -1.14 | 0.0301 | GFOD2 | NM_030819.2 | Homo sapiens glucose-fructose oxidoreductase domain containing 2 (GFOD2); mRNA >gi|18676969|dbj|AK074382.1| Homo sapiens cDNA FLJ23802 fis; clone HEP22660 |
| Ssc.19647.2.S1_at | -1.14 | 0.0342 | ADCK2 | NM_052853.3 | Homo sapiens aarF domain containing kinase 2 (ADCK2); mRNA |
| Ssc.10576.1.S1_at | -1.15 | 0.0378 | HMGN1 | NM_004965.6 | Homo sapiens high-mobility group nucleosome binding domain 1 (HMGN1); mRNA |
| Ssc.6771.1.S1_a_at | -1.15 | 0.0423 | SRI | NM_198901.1 | Homo sapiens sorcin (SRI); transcript variant 2; mRNA |
| Ssc.27323.1.A1_at | -1.15 | 0.0099 | MTA1 | NM_004689.3 | Homo sapiens metastasis associated 1 (MTA1); mRNA |
| Ssc.19242.1.A1_at | -1.15 | 0.0413 | TMEM133 | NM_032021.2 | Homo sapiens transmembrane protein 133 (TMEM133); mRNA |
| Ssc.19685.1.S1_at | -1.15 | 0.0174 | SPAST | NM_014946.3 | Homo sapiens spastin (SPAST); transcript variant 1; mRNA |
| Ssc.9684.1.S1_at | -1.15 | 0.0267 | SLC16A10 | NM_018593.3 | Homo sapiens solute carrier family 16; member 10 (aromatic amino acid transporter) (SLC16A10); mRNA >gi|44890781|gb|BC066985.1| Homo sapiens solute carrier family 16; member 10 (aromatic amino acid transporter); mRNA (cDNA clone MGC:87624 IMAGE:4827423); |
| Ssc.16893.1.S1_at | -1.15 | 0.0499 | ARFIP1 | NM_001025595.1 | Homo sapiens ADP-ribosylation factor interacting protein 1 (ARFIP1); transcript variant 1; mRNA |
| Ssc.14524.1.S1_at | -1.15 | 0.0335 | CTSL2 | NM_001333.2 | Homo sapiens cathepsin L2 (CTSL2); mRNA |
| Ssc.19040.1.A1_at | -1.15 | 0.0151 | FBXL7 | NM_012304.3 | Homo sapiens F-box and leucine-rich repeat protein 7 (FBXL7); mRNA |
| Ssc.6522.1.A1_at | -1.16 | 0.0037 | OSTM1 | NM_014028.3 | Homo sapiens osteopetrosis associated transmembrane protein 1 (OSTM1); mRNA |
| Ssc.10466.1.A1_at | -1.16 | 0.0467 | LIMCH1 | NM_001112720.1 | Homo sapiens LIM and calponin homology domains 1 (LIMCH1); transcript variant 5; mRNA |
| Ssc.17610.2.A1_at | -1.16 | 0.0144 | DNAJB6 | NM_058246.3 | Homo sapiens DnaJ (Hsp40) homolog; subfamily B; member 6 (DNAJB6); transcript variant 1; mRNA |
| Ssc.5170.1.S1_at | -1.16 | 0.0172 | DHX37 | NM_032656.2 | Homo sapiens DEAH (Asp-Glu-Ala-His) box polypeptide 37 (DHX37); mRNA |
| Ssc.1578.1.S1_at | -1.16 | 0.0057 | GGA2 | NM_015044.3 | Homo sapiens golgi associated; gamma adaptin ear containing; ARF binding protein 2 (GGA2); mRNA |
| Ssc.1849.1.A1_at | -1.16 | 0.0333 | TSEN34 | NM_001077446.1 | Homo sapiens tRNA splicing endonuclease 34 homolog (S. cerevisiae) (TSEN34); transcript variant 2; mRNA |
| Ssc.11131.1.S1_at | -1.16 | 0.0272 | VIM | NM_003380.2 | Homo sapiens vimentin (VIM); mRNA |
| Ssc.16045.2.A1_at | -1.16 | 0.0163 | FBN1 | NM_000138.3 | Homo sapiens fibrillin 1 (FBN1); mRNA |
| Ssc.25194.1.A1_at | -1.16 | 0.0312 | KIAA0562 | NM_014704.2 | Homo sapiens KIAA0562 (KIAA0562); mRNA |
| Ssc.14470.1.S2_at | -1.16 | 0.0097 | MSN | NM_002444.2 | Homo sapiens moesin (MSN); mRNA |
| Ssc.21192.3.S1_at | -1.16 | 0.0370 | MAP4K4 | NM_145686.2 | Homo sapiens mitogen-activated protein kinase kinase kinase kinase 4 (MAP4K4); transcript variant 2; mRNA |
| Ssc.14284.1.A1_at | -1.17 | 0.0149 | ZNF436 | NM_030634.2 | Homo sapiens zinc finger protein 436 (ZNF436); transcript variant 2; mRNA |
| Ssc.13250.1.A1_at | -1.17 | 0.0285 | FCHSD2 | NM_014824.2 | Homo sapiens FCH and double SH3 domains 2 (FCHSD2); mRNA |
| Ssc.9284.2.A1_at | -1.17 | 0.0415 | FAM62B | NM_020728.2 | Homo sapiens family with sequence similarity 62 (C2 domain containing) member B (FAM62B); mRNA |
| Ssc.13758.1.A1_at | -1.17 | 0.0058 | RAP2A | NM_021033.6 | Homo sapiens RAP2A; member of RAS oncogene family (RAP2A); mRNA |
| Ssc.2853.2.S1_at | -1.17 | 0.0178 | THAP11 | NM_020457.2 | Homo sapiens THAP domain containing 11 (THAP11); mRNA |
| Ssc.4233.1.S1_at | -1.17 | 0.0318 | OSBPL11 | NM_022776.4 | Homo sapiens oxysterol binding protein-like 11 (OSBPL11); mRNA |
| Ssc.11623.1.A1_at | -1.17 | 0.0426 | LAMA2 | NM_001079823.1 | Homo sapiens laminin; alpha 2 (LAMA2); transcript variant 2; mRNA |
| Ssc.25708.1.A1_at | -1.17 | 0.0026 | PTPN9 | NM_002833.2 | Homo sapiens protein tyrosine phosphatase; non-receptor type 9 (PTPN9); mRNA |
| Ssc.25002.1.S1_at | -1.17 | 0.0316 | TCF7L1 | NM_031283.1 | Homo sapiens transcription factor 7-like 1 (T-cell specific; HMG-box) (TCF7L1); mRNA >gi|11230857|dbj|AB031046.1| Homo sapiens mRNA for HMG-box transcription factor TCF-3; complete cds |
| Ssc.18181.1.A1_at | -1.17 | 0.0168 | B9D2 | NM_030578.2 | Homo sapiens B9 protein domain 2 (B9D2); mRNA >gi|33869650|gb|BC004157.2| Homo sapiens B9 protein domain 2; mRNA (cDNA clone MGC:2435 IMAGE:2819388); complete cds |
| Ssc.7610.1.S1_at | -1.17 | 0.0357 | DAZAP2 | NM_001136268.1 | Homo sapiens DAZ associated protein 2 (DAZAP2); transcript variant 5; mRNA |
| Ssc.2230.2.S1_at | -1.17 | 0.0020 | WIPF2 | NM_133264.4 | Homo sapiens WAS/WASL interacting protein family; member 2 (WIPF2); mRNA |
| Ssc.11298.1.S1_at | -1.18 | 0.0030 | PPT2 | NM_138717.1 | Homo sapiens palmitoyl-protein thioesterase 2 (PPT2); transcript variant 2; mRNA |
| Ssc.3392.1.A1_at | -1.18 | 0.0006 | ZFP36L2 | NM_006887.4 | Homo sapiens zinc finger protein 36; C3H type-like 2 (ZFP36L2); mRNA |
| Ssc.19333.3.S1_at | -1.18 | 0.0317 | TRIM27 | NM_006510.4 | Homo sapiens tripartite motif-containing 27 (TRIM27); mRNA |
| Ssc.1735.1.S1_at | -1.18 | 0.0487 | ACSL1 | NM_001995.2 | Homo sapiens acyl-CoA synthetase long-chain family member 1 (ACSL1); mRNA |
| Ssc.18195.1.S1_at | -1.18 | 0.0489 | KLHL12 | NM_021633.2 | Homo sapiens kelch-like 12 (Drosophila) (KLHL12); mRNA >gi|14042495|dbj|AK027656.1| Homo sapiens cDNA FLJ14750 fis; clone NT2RP3002948; weakly similar to RING CANAL PROTEIN |
| Ssc.26477.1.A1_at | -1.18 | 0.0100 | PALB2 | NM_024675.3 | Homo sapiens partner and localizer of BRCA2 (PALB2); mRNA |
| Ssc.6157.1.A1_at | -1.18 | 0.0320 | ZNF521 | NM_015461.1 | Homo sapiens zinc finger protein 521 (ZNF521); mRNA >gi|14041973|dbj|AK027354.1| Homo sapiens cDNA FLJ14448 fis; clone HEMBB1001482; weakly similar to ZINC FINGER PROTEIN 91 |
| Ssc.19206.1.A1_at | -1.18 | 0.0492 | PARP8 | NM_024615.2 | Homo sapiens poly (ADP-ribose) polymerase family; member 8 (PARP8); mRNA >gi|21740249|emb|AL834477.1|HSM805580 Homo sapiens mRNA; cDNA DKFZp762K2011 (from clone DKFZp762K2011) |
| Ssc.24111.1.S1_at | -1.19 | 0.0499 | ZNF684 | NM_152373.2 | Homo sapiens zinc finger protein 684 (ZNF684); mRNA >gi|21753057|dbj|AK094072.1| Homo sapiens cDNA FLJ36753 fis; clone UTERU2017761; highly similar to Zinc finger protein 684 |
| Ssc.29094.1.A1_at | -1.19 | 0.0283 | ECT2 | NM_018098.4 | Homo sapiens epithelial cell transforming sequence 2 oncogene (ECT2); mRNA |
| Ssc.3799.3.A1_a_at | -1.19 | 0.0292 | RBPMS | NM_001008712.1 | Homo sapiens RNA binding protein with multiple splicing (RBPMS); transcript variant 3; mRNA |
| Ssc.8821.1.A1_at | -1.19 | 0.0204 | MEX3C | NM_016626.3 | Homo sapiens mex-3 homolog C (C. elegans) (MEX3C); mRNA |
| Ssc.3574.1.A1_at | -1.19 | 0.0424 | MAP4K4 | NM_004834.3 | Homo sapiens mitogen-activated protein kinase kinase kinase kinase 4 (MAP4K4); transcript variant 1; mRNA |
| Ssc.9714.2.S1_at | -1.19 | 0.0252 | LMO4 | NM_006769.3 | Homo sapiens LIM domain only 4 (LMO4); mRNA |
| Ssc.17048.1.A1_at | -1.19 | 0.0000 | TMEM85 | NM_016454.2 | Homo sapiens transmembrane protein 85 (TMEM85); mRNA >gi|7106757|gb|AF151018.1|AF151018 Homo sapiens HSPC184 mRNA; complete cds |
| Ssc.10245.2.A1_a_at | -1.19 | 0.0119 | DCN | NM_133504.2 | Homo sapiens decorin (DCN); transcript variant B; mRNA |
| Ssc.1314.1.S1_at | -1.19 | 0.0241 | WDR19 | NM_025132.3 | Homo sapiens WD repeat domain 19 (WDR19); mRNA |
| Ssc.24360.1.S1_a_at | -1.19 | 0.0086 | SLC17A5 | NM_012434.4 | Homo sapiens solute carrier family 17 (anion/sugar transporter); member 5 (SLC17A5); mRNA |
| Ssc.432.1.S1_at | -1.19 | 0.0040 | BIRC5 | NM_001168.2 | Homo sapiens baculoviral IAP repeat-containing 5 (BIRC5); transcript variant 1; mRNA |
| Ssc.25853.1.A1_at | -1.19 | 0.0304 | RAB23 | NM_183227.1 | Homo sapiens RAB23; member RAS oncogene family (RAB23); transcript variant 2; mRNA |
| Ssc.3669.1.A1_a_at | -1.19 | 0.0029 | TRNAU1AP | NR_003109.1 | Homo sapiens tRNA selenocysteine 1 associated protein 1 (TRNAU1AP); transcript variant 2; transcribed RNA |
| Ssc.3574.2.A1_at | -1.19 | 0.0139 | MAP4K4 | NM_004834.3 | Homo sapiens mitogen-activated protein kinase kinase kinase kinase 4 (MAP4K4); transcript variant 1; mRNA |
| Ssc.27240.1.S1_at | -1.20 | 0.0239 | PDRG1 | NM_030815.2 | Homo sapiens p53 and DNA damage regulated 1 (PDRG1); mRNA |
| Ssc.19273.2.S1_at | -1.20 | 0.0387 | JARID1A | NM_001042603.1 | Homo sapiens jumonji; AT rich interactive domain 1A (JARID1A); transcript variant 1; mRNA |
| Ssc.20989.1.A1_at | -1.20 | 0.0348 | SLC40A1 | NM_014585.5 | Homo sapiens solute carrier family 40 (iron-regulated transporter); member 1 (SLC40A1); mRNA |
| Ssc.5715.1.S1_at | -1.20 | 0.0027 | FAM118B | NM_024556.2 | Homo sapiens family with sequence similarity 118; member B (FAM118B); mRNA |
| Ssc.7850.1.A1_at | -1.20 | 0.0164 | SMC4 | NM_005496.3 | Homo sapiens structural maintenance of chromosomes 4 (SMC4); transcript variant 1; mRNA |
| Ssc.11089.1.S1_at | -1.20 | 0.0010 | RILPL2 | NM_145058.1 | Homo sapiens Rab interacting lysosomal protein-like 2 (RILPL2); mRNA >gi|16552466|dbj|AK056934.1| Homo sapiens cDNA FLJ32372 fis; clone SALGL1000005 |
| Ssc.12356.1.A1_at | -1.20 | 0.0266 | C7orf23 | NM_024315.2 | Homo sapiens chromosome 7 open reading frame 23 (C7orf23); mRNA >gi|33877239|gb|BC002837.2| Homo sapiens chromosome 7 open reading frame 23; mRNA (cDNA clone MGC:4175 IMAGE:3634983); complete cds |
| Ssc.11079.1.A1_at | -1.20 | 0.0273 | RNASE4 | NM_194431.1 | Homo sapiens ribonuclease; RNase A family; 4 (RNASE4); transcript variant 3; mRNA |
| Ssc.16975.2.A1_at | -1.20 | 0.0173 | TANC2 | NM_025185.3 | Homo sapiens tetratricopeptide repeat; ankyrin repeat and coiled-coil containing 2 (TANC2); mRNA |
| Ssc.27893.2.S1_at | -1.20 | 0.0063 | PHF20 | NM_016436.4 | Homo sapiens PHD finger protein 20 (PHF20); mRNA |
| Ssc.4152.1.A1_at | -1.20 | 0.0364 | AKR1C1 | NM_001353.5 | Homo sapiens aldo-keto reductase family 1; member C1 (dihydrodiol dehydrogenase 1; 20-alpha (3-alpha)-hydroxysteroid dehydrogenase) (AKR1C1); mRNA |
| Ssc.30643.1.A1_at | -1.20 | 0.0377 | L3MBTL3 | NM_032438.1 | Homo sapiens l(3)mbt-like 3 (Drosophila) (L3MBTL3); transcript variant 1; mRNA |
| Ssc.2851.2.A1_at | -1.20 | 0.0444 | GEMIN5 | NM_015465.3 | Homo sapiens gem (nuclear organelle) associated protein 5 (GEMIN5); mRNA |
| Ssc.1078.2.A1_at | -1.21 | 0.0257 | CREG1 | NM_003851.2 | Homo sapiens cellular repressor of E1A-stimulated genes 1 (CREG1); mRNA |
| Ssc.27226.1.A1_at | -1.21 | 0.0319 | TTC39C | NM_001135993.1 | Homo sapiens tetratricopeptide repeat domain 39C (TTC39C); transcript variant 1; mRNA |
| Ssc.25092.1.A1_at | -1.21 | 0.0050 | MEX3D | NM_203304.3 | Homo sapiens mex-3 homolog D (C. elegans) (MEX3D); mRNA |
| Ssc.5404.1.S1_at | -1.21 | 0.0482 | MOSPD1 | NM_019556.1 | Homo sapiens motile sperm domain containing 1 (MOSPD1); mRNA >gi|6752286|emb|AL137163.1|HS473B041 Novel human gene mapping to chomosome X |
| Ssc.9035.1.A1_at | -1.21 | 0.0015 | PRSS23 | NM_007173.4 | Homo sapiens protease; serine; 23 (PRSS23); mRNA |
| Ssc.12546.1.A1_at | -1.21 | 0.0018 | LONP2 | NM_031490.2 | Homo sapiens lon peptidase 2; peroxisomal (LONP2); mRNA >gi|28804186|emb|AJ548761.1|HSA548761 Homo sapiens mRNA for for peroxisomal lon protease (LONP gene) |
| Ssc.2971.1.S1_at | -1.21 | 0.0145 | TNS1 | NM_022648.4 | Homo sapiens tensin 1 (TNS1); mRNA |
| Ssc.9498.1.S1_at | -1.21 | 0.0176 | TAX1BP3 | NM_014604.2 | Homo sapiens Tax1 (human T-cell leukemia virus type I) binding protein 3 (TAX1BP3); mRNA |
| Ssc.30916.1.A1_at | -1.21 | 0.0247 | AMIGO2 | NM_181847.3 | Homo sapiens adhesion molecule with Ig-like domain 2 (AMIGO2); mRNA |
| Ssc.11681.1.A1_at | -1.21 | 0.0069 | STX6 | NM_005819.4 | Homo sapiens syntaxin 6 (STX6); mRNA |
| Ssc.10771.1.A1_at | -1.21 | 0.0117 | UBTD2 | NM_152277.2 | Homo sapiens ubiquitin domain containing 2 (UBTD2); mRNA |
| Ssc.31127.1.A1_at | -1.21 | 0.0354 | TMTC4 | NM_032813.2 | Homo sapiens transmembrane and tetratricopeptide repeat containing 4 (TMTC4); transcript variant 1; mRNA |
| Ssc.8072.1.A1_at | -1.21 | 0.0099 | LTBP1 | NM_206943.1 | Homo sapiens latent transforming growth factor beta binding protein 1 (LTBP1); transcript variant 1; mRNA |
| Ssc.19866.1.S1_at | -1.21 | 0.0262 | CA3 | NM_005181.3 | Homo sapiens carbonic anhydrase III; muscle specific (CA3); mRNA |
| Ssc.12786.1.A1_at | -1.21 | 0.0205 | SAP30 | NM_003864.3 | Homo sapiens Sin3A-associated protein; 30kDa (SAP30); mRNA |
| Ssc.18489.2.S1_at | -1.21 | 0.0407 | MAP3K3 | NM_203351.1 | Homo sapiens mitogen-activated protein kinase kinase kinase 3 (MAP3K3); transcript variant 1; mRNA |
| Ssc.26748.1.A1_at | -1.21 | 0.0069 | HOXA9 | NM_152739.3 | Homo sapiens homeobox A9 (HOXA9); mRNA |
| Ssc.19873.2.S1_at | -1.21 | 0.0270 | CREB3L2 | NM_194071.2 | Homo sapiens cAMP responsive element binding protein 3-like 2 (CREB3L2); mRNA |
| Ssc.4848.1.S1_at | -1.22 | 0.0260 | CNN3 | NM_001839.3 | Homo sapiens calponin 3; acidic (CNN3); mRNA |
| Ssc.4345.1.S2_at | -1.22 | 0.0365 | COL4A1 | NM_001845.4 | Homo sapiens collagen; type IV; alpha 1 (COL4A1); mRNA |
| Ssc.2397.1.A1_at | -1.22 | 0.0209 | NOPE | NM_020962.1 | Homo sapiens neighbor of Punc E11 (NOPE); mRNA >gi|19570397|dbj|AB052622.1| Homo sapiens hDDM36 mRNA; complete cds |
| Ssc.16824.1.A1_at | -1.22 | 0.0440 | ITM2C | NM_001012514.1 | Homo sapiens integral membrane protein 2C (ITM2C); transcript variant 3; mRNA |
| Ssc.24928.1.S1_at | -1.22 | 0.0368 | CECR2 | NM_031413.2 | Homo sapiens cat eye syndrome chromosome region; candidate 2 (CECR2); mRNA |
| Ssc.27086.1.S1_at | -1.22 | 0.0300 | ETFDH | NM_004453.2 | Homo sapiens electron-transferring-flavoprotein dehydrogenase (ETFDH); nuclear gene encoding mitochondrial protein; mRNA |
| Ssc.9245.1.S1_at | -1.22 | 0.0038 | GALM | NM_138801.1 | Homo sapiens galactose mutarotase (aldose 1-epimerase) (GALM); mRNA >gi|17939436|gb|BC019263.1| Homo sapiens galactose mutarotase (aldose 1-epimerase); mRNA (cDNA clone MGC:3215 IMAGE:3502667); complete cds |
| Ssc.4306.1.A1_at | -1.22 | 0.0336 | MESDC1 | NM_022566.2 | Homo sapiens mesoderm development candidate 1 (MESDC1); mRNA |
| Ssc.29544.1.A1_at | -1.22 | 0.0455 | RC3H1 | NM_172071.2 | Homo sapiens ring finger and CCCH-type zinc finger domains 1 (RC3H1); mRNA |
| Ssc.13549.1.A1_at | -1.22 | 0.0002 | STARD3NL | NM_032016.2 | Homo sapiens STARD3 N-terminal like (STARD3NL); mRNA >gi|25136919|emb|AJ492267.1|HSA492267 Homo sapiens mRNA for MLN64 N-terminal homolog protein (MENTHO gene) |
| Ssc.7661.2.S1_at | -1.22 | 0.0463 | HABP4 | NM_014282.2 | Homo sapiens hyaluronan binding protein 4 (HABP4); mRNA |
| Ssc.19823.1.A1_at | -1.22 | 0.0066 | ABLIM1 | NM_001003408.1 | Homo sapiens actin binding LIM protein 1 (ABLIM1); transcript variant 3; mRNA |
| Ssc.12617.1.S1_at | -1.22 | 0.0368 | COL5A3 | NM_015719.3 | Homo sapiens collagen; type V; alpha 3 (COL5A3); mRNA |
| Ssc.26296.1.S1_at | -1.23 | 0.0023 | TRAM2 | NM_012288.3 | Homo sapiens translocation associated membrane protein 2 (TRAM2); mRNA |
| Ssc.21869.1.S1_at | -1.23 | 0.0202 | SKAP2 | NM_003930.3 | Homo sapiens src kinase associated phosphoprotein 2 (SKAP2); mRNA |
| Ssc.1466.1.A1_at | -1.23 | 0.0461 | AURKA | NM_003600.2 | Homo sapiens aurora kinase A (AURKA); transcript variant 2; mRNA |
| Ssc.30414.1.A1_at | -1.23 | 0.0049 | KIAA2013 | NM_138346.1 | Homo sapiens KIAA2013 (KIAA2013); mRNA |
| Ssc.10184.1.S1_at | -1.23 | 0.0127 | S100PBP | NM_022753.2 | Homo sapiens S100P binding protein (S100PBP); transcript variant 1; mRNA |
| Ssc.9611.1.A1_at | -1.23 | 0.0066 | SESN1 | NM_014454.1 | Homo sapiens sestrin 1 (SESN1); mRNA >gi|4092862|gb|AF033122.1|AF033122 Homo sapiens non-p53 regulated PA26-T1 nuclear protein (PA26) mRNA; complete cds |
| Ssc.14997.1.A1_at | -1.23 | 0.0020 | TRIM27 | NM_006510.4 | Homo sapiens tripartite motif-containing 27 (TRIM27); mRNA |
| Ssc.1121.1.S1_at | -1.23 | 0.0336 | PDK4 | NM_002612.3 | Homo sapiens pyruvate dehydrogenase kinase; isozyme 4 (PDK4); mRNA |
| Ssc.5122.1.A1_at | -1.23 | 0.0165 | PIM2 | NM_006875.3 | Homo sapiens pim-2 oncogene (PIM2); mRNA |
| Ssc.10199.2.S1_a_at | -1.24 | 0.0482 | DTNBP1 | NM_183040.1 | Homo sapiens dystrobrevin binding protein 1 (DTNBP1); transcript variant 2; mRNA |
| Ssc.8312.1.A1_at | -1.24 | 0.0003 | CDYL | NM_170751.1 | Homo sapiens chromodomain protein; Y-like (CDYL); transcript variant 2; mRNA |
| Ssc.7036.1.A1_at | -1.24 | 0.0101 | ZNF32 | NM_006973.2 | Homo sapiens zinc finger protein 32 (ZNF32); transcript variant 1; mRNA |
| Ssc.8330.1.S1_at | -1.24 | 0.0131 | RGS5 | NM_003617.2 | Homo sapiens regulator of G-protein signaling 5 (RGS5); mRNA |
| Ssc.22305.1.A1_at | -1.24 | 0.0238 | DAPK1 | NM_004938.2 | Homo sapiens death-associated protein kinase 1 (DAPK1); mRNA |
| Ssc.5403.1.S1_at | -1.24 | 0.0423 | ZNF703 | NM_025069.1 | Homo sapiens zinc finger protein 703 (ZNF703); mRNA >gi|10436728|dbj|AK024361.1| Homo sapiens cDNA FLJ14299 fis; clone PLACE1010310; weakly similar to SPIDROIN 2 |
| Ssc.8368.1.A1_at | -1.24 | 0.0001 | PDZD8 | NM_173791.3 | Homo sapiens PDZ domain containing 8 (PDZD8); mRNA |
| Ssc.18206.1.S1_at | -1.24 | 0.0065 | BACE2 | NM_012105.3 | Homo sapiens beta-site APP-cleaving enzyme 2 (BACE2); transcript variant a; mRNA |
| Ssc.20273.1.S1_at | -1.24 | 0.0100 | ZFP36L1 | NM_004926.2 | Homo sapiens zinc finger protein 36; C3H type-like 1 (ZFP36L1); mRNA |
| Ssc.5604.1.S1_at | -1.24 | 0.0137 | PROS1 | NM_000313.2 | Homo sapiens protein S (alpha) (PROS1); mRNA |
| Ssc.7529.1.S1_at | -1.24 | 0.0118 | RPS23 | NM_001025.4 | Homo sapiens ribosomal protein S23 (RPS23); mRNA |
| Ssc.4913.1.A1_at | -1.24 | 0.0056 | ENPP1 | NM_006208.2 | Homo sapiens ectonucleotide pyrophosphatase/phosphodiesterase 1 (ENPP1); mRNA |
| Ssc.9547.1.S1_at | -1.24 | 0.0090 | SLC30A5 | NM_022902.2 | Homo sapiens solute carrier family 30 (zinc transporter); member 5 (SLC30A5); transcript variant 1; mRNA >gi|19744303|gb|AF461760.1| Homo sapiens zinc transporter 5 (ZNT5) mRNA; complete cds |
| Ssc.29216.1.A1_at | -1.24 | 0.0069 | LAYN | NM_178834.3 | Homo sapiens layilin (LAYN); mRNA |
| Ssc.8529.1.A1_at | -1.24 | 0.0104 | ZFP36L1 | NM_004926.2 | Homo sapiens zinc finger protein 36; C3H type-like 1 (ZFP36L1); mRNA |
| Ssc.9087.1.A1_at | -1.25 | 0.0390 | MGC16121 | XM_001715872.1 | PREDICTED: Homo sapiens hypothetical protein MGC16121 (MGC16121); mRNA |
| Ssc.11167.1.A1_at | -1.25 | 0.0002 | RMND5B | NM_022762.3 | Homo sapiens required for meiotic nuclear division 5 homolog B (S. cerevisiae) (RMND5B); mRNA >gi|33869728|gb|BC009911.2| Homo sapiens required for meiotic nuclear division 5 homolog B (S. cerevisiae); mRNA (cDNA clone MGC:2688 IMAGE:2820100); complete cd |
| Ssc.3799.3.A1_at | -1.25 | 0.0153 | RBPMS | NM_001008710.1 | Homo sapiens RNA binding protein with multiple splicing (RBPMS); transcript variant 1; mRNA |
| Ssc.17330.1.A1_at | -1.25 | 0.0394 | NSMCE4A | NM_017615.1 | Homo sapiens non-SMC element 4 homolog A (S. cerevisiae) (NSMCE4A); mRNA >gi|7019814|dbj|AK000010.1| Homo sapiens cDNA FLJ20003 fis; clone ADKA01794 |
| Ssc.20414.1.S1_at | -1.25 | 0.0129 | PLOD3 | NM_001084.4 | Homo sapiens procollagen-lysine; 2-oxoglutarate 5-dioxygenase 3 (PLOD3); mRNA |
| Ssc.5282.1.S1_at | -1.25 | 0.0304 | CREB3L4 | NM_130898.2 | Homo sapiens cAMP responsive element binding protein 3-like 4 (CREB3L4); mRNA >gi|27260906|dbj|AB052778.1| Homo sapiens hJAL mRNA; complete cds |
| Ssc.1078.1.A1_at | -1.25 | 0.0060 | CREG1 | NM_003851.2 | Homo sapiens cellular repressor of E1A-stimulated genes 1 (CREG1); mRNA |
| Ssc.23026.1.A1_at | -1.25 | 0.0375 | C6orf168 | NM_032511.2 | Homo sapiens chromosome 6 open reading frame 168 (C6orf168); mRNA |
| Ssc.26271.1.S1_at | -1.25 | 0.0060 | EBNA1BP2 | NM_006824.1 | Homo sapiens EBNA1 binding protein 2 (EBNA1BP2); mRNA >gi|1835785|gb|U86602.1|HSU86602 Human nucleolar protein p40 mRNA; complete cds |
| Ssc.8072.2.A1_at | -1.25 | 0.0090 | LTBP1 | NM_206943.1 | Homo sapiens latent transforming growth factor beta binding protein 1 (LTBP1); transcript variant 1; mRNA |
| Ssc.7790.1.S1_at | -1.26 | 0.0091 | WDR51B | NM_172240.1 | Homo sapiens WD repeat domain 51B (WDR51B); mRNA >gi|22760435|dbj|AK074772.1| Homo sapiens cDNA FLJ90291 fis; clone NT2RP1001031; weakly similar to VEGETATIBLE INCOMPATIBILITY PROTEIN HET-E-1 |
| Ssc.19192.2.A1_at | -1.26 | 0.0027 | C20orf108 | NM_080821.2 | Homo sapiens chromosome 20 open reading frame 108 (C20orf108); mRNA |
| Ssc.13494.1.A1_at | -1.26 | 0.0061 | LHFP | NM_005780.2 | Homo sapiens lipoma HMGIC fusion partner (LHFP); mRNA |
| Ssc.28050.1.A1_at | -1.26 | 0.0487 | HIST2H2BF | NM_001024599.2 | Homo sapiens histone cluster 2; H2bf (HIST2H2BF); mRNA >gi|84570000|gb|BC110793.1| Homo sapiens histone cluster 2; H2bf; mRNA (cDNA clone MGC:131639 IMAGE:5224812); complete cds |
| Ssc.11787.2.A1_at | -1.26 | 0.0179 | RASSF2 | NM_014737.2 | Homo sapiens Ras association (RalGDS/AF-6) domain family member 2 (RASSF2); transcript variant 1; mRNA |
| Ssc.1116.1.S1_at | -1.26 | 0.0129 | CPD | NM_001304.3 | Homo sapiens carboxypeptidase D (CPD); mRNA |
| Ssc.7070.1.S1_at | -1.26 | 0.0075 | SESN1 | NM_014454.1 | Homo sapiens sestrin 1 (SESN1); mRNA >gi|4092862|gb|AF033122.1|AF033122 Homo sapiens non-p53 regulated PA26-T1 nuclear protein (PA26) mRNA; complete cds |
| Ssc.16714.1.S1_at | -1.26 | 0.0329 | REEP4 | NM_025232.2 | Homo sapiens receptor accessory protein 4 (REEP4); mRNA >gi|15341768|gb|BC013048.1| Homo sapiens receptor accessory protein 4; mRNA (cDNA clone MGC:9377 IMAGE:3863838); complete cds |
| Ssc.12229.1.S1_at | -1.26 | 0.0485 | CKS2 | NM_001827.1 | Homo sapiens CDC28 protein kinase regulatory subunit 2 (CKS2); mRNA >gi|29978|emb|X54942.1|HSCKSHS2 H.sapiens ckshs2 mRNA for Cks1 protein homologue |
| Ssc.29939.1.S1_at | -1.26 | 0.0240 | FLJ42709 | NR_021491.1 | Homo sapiens hypothetical gene supported by AK124699 (FLJ42709); non-coding RNA |
| Ssc.6270.1.S1_at | -1.26 | 0.0075 | IMP4 | NM_033416.1 | Homo sapiens IMP4; U3 small nucleolar ribonucleoprotein; homolog (yeast) (IMP4); mRNA >gi|14603152|gb|BC010042.1| Homo sapiens IMP4; U3 small nucleolar ribonucleoprotein; homolog (yeast); mRNA (cDNA clone MGC:19606 IMAGE:3629513); complete cds |
| Ssc.1367.1.A1_at | -1.27 | 0.0053 | SPAG1 | NM_172218.1 | Homo sapiens sperm associated antigen 1 (SPAG1); transcript variant 2; mRNA |
| Ssc.14025.1.A1_at | -1.27 | 0.0302 | LEF1 | NM_001130713.1 | Homo sapiens lymphoid enhancer-binding factor 1 (LEF1); transcript variant 2; mRNA |
| Ssc.5953.1.S1_at | -1.27 | 0.0000 | C14orf119 | NM_017924.2 | Homo sapiens chromosome 14 open reading frame 119 (C14orf119); mRNA >gi|16307113|gb|BC009645.1| Homo sapiens chromosome 14 open reading frame 119; mRNA (cDNA clone MGC:4950 IMAGE:3458006); complete cds |
| Ssc.11663.1.A1_at | -1.27 | 0.0128 | TRIM13 | NM_052811.2 | Homo sapiens tripartite motif-containing 13 (TRIM13); transcript variant 2; mRNA |
| Ssc.3931.1.S1_at | -1.27 | 0.0285 | FHL3 | NM_004468.3 | Homo sapiens four and a half LIM domains 3 (FHL3); mRNA |
| Ssc.8904.1.A1_at | -1.27 | 0.0499 | ADAMTSL3 | NM_207517.2 | Homo sapiens ADAMTS-like 3 (ADAMTSL3); mRNA |
| Ssc.11415.1.A1_at | -1.27 | 0.0012 | LAMA2 | NM_001079823.1 | Homo sapiens laminin; alpha 2 (LAMA2); transcript variant 2; mRNA |
| Ssc.18187.2.S1_at | -1.27 | 0.0023 | CCNE1 | NM_057182.1 | Homo sapiens cyclin E1 (CCNE1); transcript variant 2; mRNA |
| Ssc.13553.1.A1_at | -1.27 | 0.0056 | GNA14 | NM_004297.2 | Homo sapiens guanine nucleotide binding protein (G protein); alpha 14 (GNA14); mRNA >gi|24081078|gb|BC027886.1| Homo sapiens guanine nucleotide binding protein (G protein); alpha 14; mRNA (cDNA clone MGC:34487 IMAGE:5221889); complete cds |
| Ssc.20424.1.S1_at | -1.27 | 0.0032 | SIAH2 | NM_005067.5 | Homo sapiens seven in absentia homolog 2 (Drosophila) (SIAH2); mRNA |
| Ssc.6578.1.S1_at | -1.27 | 0.0241 | TBX3 | NM_016569.3 | Homo sapiens T-box 3 (TBX3); transcript variant 2; mRNA |
| Ssc.9560.1.S1_at | -1.27 | 0.0004 | RPL11 | NM_000975.2 | Homo sapiens ribosomal protein L11 (RPL11); mRNA |
| Ssc.14227.1.A1_at | -1.27 | 0.0034 | POGK | NM_017542.3 | Homo sapiens pogo transposable element with KRAB domain (POGK); mRNA |
| Ssc.17615.1.S1_at | -1.28 | 0.0016 | ATP1B1 | NM_001677.3 | Homo sapiens ATPase; Na+/K+ transporting; beta 1 polypeptide (ATP1B1); transcript variant 1; mRNA |
| Ssc.27616.1.S1_at | -1.28 | 0.0027 | CDK2 | NM_001798.3 | Homo sapiens cyclin-dependent kinase 2 (CDK2); transcript variant 1; mRNA |
| Ssc.20424.3.S1_a_at | -1.28 | 0.0062 | SIAH2 | NM_005067.5 | Homo sapiens seven in absentia homolog 2 (Drosophila) (SIAH2); mRNA |
| Ssc.18603.1.A1_at | -1.28 | 0.0212 | G0S2 | NM_015714.3 | Homo sapiens G0/G1switch 2 (G0S2); mRNA |
| Ssc.19613.3.A1_at | -1.28 | 0.0402 | ARMC6 | NM_033415.2 | Homo sapiens armadillo repeat containing 6 (ARMC6); mRNA |
| Ssc.7136.1.A1_at | -1.28 | 0.0309 | CDC2L6 | NM_015076.3 | Homo sapiens cell division cycle 2-like 6 (CDK8-like) (CDC2L6); mRNA |
| Ssc.5713.1.S1_at | -1.29 | 0.0084 | MMP2 | NM_004530.4 | Homo sapiens matrix metallopeptidase 2 (gelatinase A; 72kDa gelatinase; 72kDa type IV collagenase) (MMP2); transcript variant 1; mRNA |
| Ssc.24969.1.S1_at | -1.29 | 0.0095 | KRCC1 | NM_016618.1 | Homo sapiens lysine-rich coiled-coil 1 (KRCC1); mRNA >gi|7582277|gb|AF208845.1|AF208845 Homo sapiens BM-003 mRNA; complete cds |
| Ssc.21360.1.A1_at | -1.29 | 0.0137 | CLSPN | NM_022111.2 | Homo sapiens claspin homolog (Xenopus laevis) (CLSPN); mRNA |
| Ssc.6512.1.S1_at | -1.29 | 0.0204 | SCARA5 | NM_173833.4 | Homo sapiens scavenger receptor class A; member 5 (putative) (SCARA5); mRNA |
| Ssc.5800.1.A1_at | -1.29 | 0.0095 | BEND7 | NM_001100912.1 | Homo sapiens BEN domain containing 7 (BEND7); transcript variant 2; mRNA |
| Ssc.19675.1.S1_at | -1.29 | 0.0011 | RACGAP1 | NM_001126104.1 | Homo sapiens Rac GTPase activating protein 1 (RACGAP1); transcript variant 3; mRNA |
| Ssc.19691.1.S1_at | -1.29 | 0.0117 | PLA2G7 | NM_005084.3 | Homo sapiens phospholipase A2; group VII (platelet-activating factor acetylhydrolase; plasma) (PLA2G7); mRNA |
| Ssc.9693.1.A1_at | -1.30 | 0.0115 | TMEM100 | NM_001099640.1 | Homo sapiens transmembrane protein 100 (TMEM100); transcript variant 1; mRNA |
| Ssc.26290.1.S1_at | -1.30 | 0.0138 | ITGB5 | NM_002213.3 | Homo sapiens integrin; beta 5 (ITGB5); mRNA >gi|33869594|gb|BC006541.2| Homo sapiens integrin; beta 5; mRNA (cDNA clone MGC:2338 IMAGE:2958666); complete cds |
| Ssc.25678.1.S1_at | -1.30 | 0.0297 | LOC100130890 | XM_001721586.1 | PREDICTED: Homo sapiens similar to hCG2030844; transcript variant 1 (LOC100130890); mRNA |
| Ssc.4580.1.A1_at | -1.30 | 0.0330 | UBTD2 | NM_152277.2 | Homo sapiens ubiquitin domain containing 2 (UBTD2); mRNA |
| Ssc.30401.1.A1_at | -1.30 | 0.0031 | KDELC2 | NM_153705.4 | Homo sapiens KDEL (Lys-Asp-Glu-Leu) containing 2 (KDELC2); mRNA |
| Ssc.28673.1.S1_at | -1.31 | 0.0273 | CXCR7 | NM_020311.2 | Homo sapiens chemokine (C-X-C motif) receptor 7 (CXCR7); mRNA |
| Ssc.19571.2.S1_at | -1.31 | 0.0204 | ABHD4 | NM_022060.2 | Homo sapiens abhydrolase domain containing 4 (ABHD4); mRNA |
| Ssc.16934.1.S1_at | -1.31 | 0.0089 | FDXR | NM_024417.2 | Homo sapiens ferredoxin reductase (FDXR); nuclear gene encoding mitochondrial protein; transcript variant 1; mRNA |
| Ssc.3394.1.A1_at | -1.31 | 0.0347 | CDR2 | NM_001802.1 | Homo sapiens cerebellar degeneration-related protein 2; 62kDa (CDR2); mRNA |
| Ssc.4679.1.S1_at | -1.31 | 0.0059 | ANG | NM_001145.4 | Homo sapiens angiogenin; ribonuclease; RNase A family; 5 (ANG); transcript variant 1; mRNA |
| Ssc.21605.2.S1_at | -1.32 | 0.0383 | NUSAP1 | NM_016359.3 | Homo sapiens nucleolar and spindle associated protein 1 (NUSAP1); transcript variant 1; mRNA |
| Ssc.9896.1.A1_at | -1.32 | 0.0478 | ST8SIA1 | NM_003034.3 | Homo sapiens ST8 alpha-N-acetyl-neuraminide alpha-2;8-sialyltransferase 1 (ST8SIA1); mRNA |
| Ssc.5169.1.A1_at | -1.32 | 0.0451 | HTRA3 | NM_053044.2 | Homo sapiens HtrA serine peptidase 3 (HTRA3); mRNA >gi|21706740|gb|BC034390.1| Homo sapiens HtrA serine peptidase 3; mRNA (cDNA clone MGC:35339 IMAGE:5180585); complete cds |
| Ssc.1086.1.A1_at | -1.33 | 0.0388 | MFAP5 | NM_003480.2 | Homo sapiens microfibrillar associated protein 5 (MFAP5); mRNA |
| Ssc.30871.1.A1_at | -1.33 | 0.0499 | TP53INP1 | NM_001135733.1 | Homo sapiens tumor protein p53 inducible nuclear protein 1 (TP53INP1); transcript variant 2; mRNA |
| Ssc.28616.1.S1_at | -1.33 | 0.0176 | FBXL21 | NM_012159.2 | Homo sapiens F-box and leucine-rich repeat protein 21 (FBXL21); mRNA |
| Ssc.18892.1.A1_at | -1.33 | 0.0496 | TNNC2 | NM_003279.2 | Homo sapiens troponin C type 2 (fast) (TNNC2); mRNA |
| Ssc.21626.2.S1_at | -1.34 | 0.0448 | GAS1 | NM_002048.2 | Homo sapiens growth arrest-specific 1 (GAS1); mRNA |
| Ssc.17974.1.A1_at | -1.34 | 0.0180 | TTC9 | NM_015351.1 | Homo sapiens tetratricopeptide repeat domain 9 (TTC9); mRNA |
| Ssc.5708.1.A1_at | -1.34 | 0.0089 | PLSCR4 | NM_001128305.1 | Homo sapiens phospholipid scramblase 4 (PLSCR4); transcript variant 3; mRNA |
| Ssc.24873.1.S1_at | -1.34 | 0.0262 | CBX2 | NM_005189.1 | Homo sapiens chromobox homolog 2 (Pc class homolog; Drosophila) (CBX2); transcript variant 1; mRNA |
| Ssc.13414.1.A1_at | -1.34 | 0.0006 | L3MBTL3 | NM_032438.1 | Homo sapiens l(3)mbt-like 3 (Drosophila) (L3MBTL3); transcript variant 1; mRNA |
| Ssc.7382.1.A1_s_at | -1.35 | 0.0304 | CCDC52 | NM_144718.3 | Homo sapiens coiled-coil domain containing 52 (CCDC52); mRNA |
| Ssc.21928.1.A1_at | -1.35 | 0.0045 | PM20D2 | NM_001010853.1 | Homo sapiens peptidase M20 domain containing 2 (PM20D2); mRNA |
| Ssc.5587.1.A1_at | -1.35 | 0.0000 | NID1 | NM_002508.2 | Homo sapiens nidogen 1 (NID1); mRNA |
| Ssc.373.1.S1_at | -1.35 | 0.0295 | LOC100133713 | XM_001715885.1 | PREDICTED: Homo sapiens hypothetical protein LOC100133713 (LOC100133713); mRNA |
| Ssc.1974.1.A1_at | -1.35 | 0.0217 | TICAM2 | NM_021649.4 | Homo sapiens toll-like receptor adaptor molecule 2 (TICAM2); mRNA |
| Ssc.5129.1.S1_at | -1.36 | 0.0014 | MAD2L1 | NM_002358.3 | Homo sapiens MAD2 mitotic arrest deficient-like 1 (yeast) (MAD2L1); mRNA |
| Ssc.6145.1.A1_at | -1.36 | 0.0153 | CYBRD1 | NM_024843.3 | Homo sapiens cytochrome b reductase 1 (CYBRD1); transcript variant 1; mRNA |
| Ssc.28059.1.A1_at | -1.36 | 0.0003 | RFFL | NM_057178.3 | Homo sapiens ring finger and FYVE-like domain containing 1 (RFFL); transcript variant 1; mRNA |
| Ssc.24037.1.S1_at | -1.36 | 0.0040 | KIAA1211 | NM_020722.1 | Homo sapiens KIAA1211 protein (KIAA1211); mRNA |
| Ssc.4141.1.A1_at | -1.37 | 0.0241 | KCTD12 | NM_138444.3 | Homo sapiens potassium channel tetramerisation domain containing 12 (KCTD12); mRNA |
| Ssc.24731.1.A1_at | -1.37 | 0.0007 | MEX3B | NM_032246.3 | Homo sapiens mex-3 homolog B (C. elegans) (MEX3B); mRNA >gi|47077364|dbj|AK131424.1| Homo sapiens cDNA FLJ16544 fis; clone OCBBF3003761 |
| Ssc.8144.1.A1_at | -1.37 | 0.0379 | SLAIN1 | NM_001040153.2 | Homo sapiens SLAIN motif family; member 1 (SLAIN1); transcript variant 1; mRNA |
| Ssc.639.1.A1_at | -1.37 | 0.0412 | GEM | NM_181702.1 | Homo sapiens GTP binding protein overexpressed in skeletal muscle (GEM); transcript variant 2; mRNA |
| Ssc.10264.1.A1_at | -1.37 | 0.0498 | PTPN3 | NM_002829.2 | Homo sapiens protein tyrosine phosphatase; non-receptor type 3 (PTPN3); mRNA |
| Ssc.24889.1.S1_at | -1.37 | 0.0429 | ALOX12 | NM_000697.2 | Homo sapiens arachidonate 12-lipoxygenase (ALOX12); mRNA |
| Ssc.24807.1.A1_at | -1.37 | 0.0163 | SLC38A4 | NM_018018.3 | Homo sapiens solute carrier family 38; member 4 (SLC38A4); mRNA |
| Ssc.6752.1.S1_at | -1.37 | 0.0043 | BIVM | NM_017693.2 | Homo sapiens basic; immunoglobulin-like variable motif containing (BIVM); mRNA >gi|21305830|gb|AF411385.1| Homo sapiens basic; immunoglobulin-like variable motif-containing protein (BIVM) mRNA; complete cds |
| Ssc.383.1.S1_at | -1.38 | 0.0315 | HOPX | NM_139212.2 | Homo sapiens HOP homeobox (HOPX); transcript variant 3; mRNA |
| Ssc.7158.1.A1_a_at | -1.38 | 0.0002 | CAPNS1 | NM_001749.2 | Homo sapiens calpain; small subunit 1 (CAPNS1); transcript variant 1; mRNA |
| Ssc.11815.1.A1_s_at | -1.38 | 0.0000 | LAMA2 | NM_001079823.1 | Homo sapiens laminin; alpha 2 (LAMA2); transcript variant 2; mRNA |
| Ssc.7116.1.A1_at | -1.38 | 0.0449 | NT5C3 | NM_001002009.1 | Homo sapiens 5'-nucleotidase; cytosolic III (NT5C3); transcript variant 2; mRNA |
| Ssc.4899.1.S1_at | -1.38 | 0.0005 | SRPX2 | NM_014467.2 | Homo sapiens sushi-repeat-containing protein; X-linked 2 (SRPX2); mRNA |
| Ssc.4770.1.A1_at | -1.39 | 0.0133 | RNU6-1 | NR_004394.1 | Homo sapiens RNA; U6 small nuclear 1 (RNU6-1); non-coding RNA |
| Ssc.26552.1.A1_at | -1.39 | 0.0216 | ADAMTS17 | NM_139057.2 | Homo sapiens ADAM metallopeptidase with thrombospondin type 1 motif; 17 (ADAMTS17); mRNA |
| Ssc.24441.2.S1_a_at | -1.40 | 0.0066 | NPAS2 | NM_002518.3 | Homo sapiens neuronal PAS domain protein 2 (NPAS2); mRNA |
| Ssc.12842.1.S1_at | -1.40 | 0.0010 | CAV1 | NM_001753.3 | Homo sapiens caveolin 1; caveolae protein; 22kDa (CAV1); mRNA |
| Ssc.27508.1.A1_at | -1.41 | 0.0001 | SATB2 | NM_015265.2 | Homo sapiens SATB homeobox 2 (SATB2); mRNA |
| Ssc.19873.1.S1_a_at | -1.41 | 0.0020 | CREB3L2 | NM_194071.2 | Homo sapiens cAMP responsive element binding protein 3-like 2 (CREB3L2); mRNA |
| Ssc.5453.1.A1_at | -1.41 | 0.0042 | QPCT | NM_012413.3 | Homo sapiens glutaminyl-peptide cyclotransferase (QPCT); mRNA |
| Ssc.3020.1.A1_at | -1.42 | 0.0000 | IQGAP2 | NM_006633.2 | Homo sapiens IQ motif containing GTPase activating protein 2 (IQGAP2); mRNA |
| Ssc.17330.2.A1_at | -1.42 | 0.0002 | NSMCE4A | NM_017615.1 | Homo sapiens non-SMC element 4 homolog A (S. cerevisiae) (NSMCE4A); mRNA >gi|7019814|dbj|AK000010.1| Homo sapiens cDNA FLJ20003 fis; clone ADKA01794 |
| Ssc.30963.1.A1_at | -1.42 | 0.0365 | SLC6A4 | NM_001045.3 | Homo sapiens solute carrier family 6 (neurotransmitter transporter; serotonin); member 4 (SLC6A4); mRNA |
| Ssc.16570.1.S1_at | -1.43 | 0.0300 | ELN | NM_001081752.1 | Homo sapiens elastin (ELN); transcript variant 2; mRNA |
| Ssc.19358.1.S1_at | -1.44 | 0.0275 | ZDHHC9 | NM_016032.2 | Homo sapiens zinc finger; DHHC-type containing 9 (ZDHHC9); transcript variant 1; mRNA |
| Ssc.11038.1.A1_at | -1.44 | 0.0005 | FBLN5 | NM_006329.3 | Homo sapiens fibulin 5 (FBLN5); mRNA |
| Ssc.14243.1.S1_at | -1.45 | 0.0418 | CCNB1 | NM_031966.2 | Homo sapiens cyclin B1 (CCNB1); mRNA |
| Ssc.23222.1.S1_at | -1.45 | 0.0065 | PDXP | NM_020315.4 | Homo sapiens pyridoxal (pyridoxine; vitamin B6) phosphatase (PDXP); mRNA >gi|40674426|gb|BC064922.1| Homo sapiens pyridoxal (pyridoxine; vitamin B6) phosphatase; mRNA (cDNA clone MGC:74719 IMAGE:6141538); complete cds |
| Ssc.29259.1.A1_at | -1.46 | 0.0026 | ZNF567 | NM_152603.2 | Homo sapiens zinc finger protein 567 (ZNF567); mRNA >gi|34192436|gb|BC033849.2| Homo sapiens zinc finger protein 567; mRNA (cDNA clone MGC:45586 IMAGE:4472579); complete cds |
| Ssc.10382.1.A1_at | -1.46 | 0.0145 | MYO5B | NM_001080467.1 | Homo sapiens myosin VB (MYO5B); mRNA |
| Ssc.8267.1.A1_at | -1.46 | 0.0012 | AK5 | NM_012093.2 | Homo sapiens adenylate kinase 5 (AK5); transcript variant 2; mRNA |
| Ssc.25637.1.S1_at | -1.47 | 0.0491 | UPP2 | NM_001135098.1 | Homo sapiens uridine phosphorylase 2 (UPP2); transcript variant 2; mRNA |
| Ssc.1810.1.A1_at | -1.48 | 0.0027 | HOXD3 | NM_006898.4 | Homo sapiens homeobox D3 (HOXD3); mRNA |
| Ssc.26492.1.A1_at | -1.48 | 0.0037 | KLF11 | NM_003597.4 | Homo sapiens Kruppel-like factor 11 (KLF11); mRNA |
| Ssc.5987.1.A1_at | -1.48 | 0.0162 | RHPN2 | NM_033103.3 | Homo sapiens rhophilin; Rho GTPase binding protein 2 (RHPN2); mRNA |
| Ssc.19694.1.S1_at | -1.49 | 0.0012 | GPX3 | NM_002084.3 | Homo sapiens glutathione peroxidase 3 (plasma) (GPX3); mRNA |
| Ssc.19213.1.S1_at | -1.50 | 0.0321 | TRIML2 | NM_173553.1 | Homo sapiens tripartite motif family-like 2 (TRIML2); mRNA >gi|21758745|dbj|AK098667.1| Homo sapiens cDNA FLJ25801 fis; clone TST07120 |
| Ssc.11844.1.A1_at | -1.51 | 0.0444 | ARHGAP28 | NM_001010000.1 | Homo sapiens Rho GTPase activating protein 28 (ARHGAP28); transcript variant 1; mRNA |
| Ssc.13696.1.A1_at | -1.51 | 0.0126 | DDIT4L | NM_145244.2 | Homo sapiens DNA-damage-inducible transcript 4-like (DDIT4L); mRNA >gi|34189377|gb|BC013592.2| Homo sapiens DNA-damage-inducible transcript 4-like; mRNA (cDNA clone MGC:9960 IMAGE:3877854); complete cds |
| Ssc.9483.1.A1_s_at | -1.51 | 0.0018 | OSBPL6 | NM_145739.1 | Homo sapiens oxysterol binding protein-like 6 (OSBPL6); transcript variant 2; mRNA |
| Ssc.26113.1.S1_at | -1.52 | 0.0064 | FAM134B | NM_019000.3 | Homo sapiens family with sequence similarity 134; member B (FAM134B); transcript variant 2; mRNA |
| Ssc.7594.1.A1_at | -1.52 | 0.0007 | DEPDC1B | NM_018369.1 | Homo sapiens DEP domain containing 1B (DEPDC1B); mRNA |
| Ssc.26361.1.A1_at | -1.53 | 0.0109 | ZNF12 | NM_006956.2 | Homo sapiens zinc finger protein 12 (ZNF12); transcript variant 2; mRNA |
| Ssc.19488.1.A1_at | -1.54 | 0.0345 | DDX50 | NM_024045.1 | Homo sapiens DEAD (Asp-Glu-Ala-Asp) box polypeptide 50 (DDX50); mRNA >gi|12653020|gb|BC000272.1| Homo sapiens DEAD (Asp-Glu-Ala-Asp) box polypeptide 50; mRNA (cDNA clone MGC:3199 IMAGE:3357684); complete cds |
| Ssc.25553.1.S1_at | -1.54 | 0.0024 | PTPN3 | NM_002829.2 | Homo sapiens protein tyrosine phosphatase; non-receptor type 3 (PTPN3); mRNA |
| Ssc.15685.1.A1_at | -1.58 | 0.0040 | LOC100131218 | XM_001714467.1 | PREDICTED: Homo sapiens similar to NADPH-dependent FMN and FAD containing oxidoreductase (LOC100131218); partial mRNA |
| Ssc.3739.1.S1_at | -1.60 | 0.0013 | PCSK6 | NM_002570.3 | Homo sapiens proprotein convertase subtilisin/kexin type 6 (PCSK6); transcript variant 1; mRNA |
| Ssc.11264.1.A1_at | -1.60 | 0.0466 | MCM5 | NM_006739.3 | Homo sapiens minichromosome maintenance complex component 5 (MCM5); mRNA |
| Ssc.3319.1.S1_at | -1.61 | 0.0074 | ITIH1 | NM_002215.2 | Homo sapiens inter-alpha (globulin) inhibitor H1 (ITIH1); mRNA |
| Ssc.18522.1.A1_at | -1.61 | 0.0006 | MLYCD | NM_012213.2 | Homo sapiens malonyl-CoA decarboxylase (MLYCD); nuclear gene encoding mitochondrial protein; mRNA >gi|30962891|gb|BC052592.1| Homo sapiens malonyl-CoA decarboxylase; mRNA (cDNA clone MGC:59795 IMAGE:6421051); complete cds |
| Ssc.23981.1.A1_at | -1.61 | 0.0017 | GALNT12 | NM_024642.3 | Homo sapiens UDP-N-acetyl-alpha-D-galactosamine:polypeptide N-acetylgalactosaminyltransferase 12 (GalNAc-T12) (GALNT12); mRNA |
| Ssc.29168.1.A1_at | -1.62 | 0.0006 | PLA2G4A | NM_024420.2 | Homo sapiens phospholipase A2; group IVA (cytosolic; calcium-dependent) (PLA2G4A); mRNA |
| Ssc.6813.1.A1_at | -1.62 | 0.0444 | CDH9 | NM_016279.3 | Homo sapiens cadherin 9; type 2 (T1-cadherin) (CDH9); mRNA |
| Ssc.7530.1.S1_at | -1.63 | 0.0431 | ANKS4B | NM_145865.2 | Homo sapiens ankyrin repeat and sterile alpha motif domain containing 4B (ANKS4B); mRNA |
| Ssc.26113.2.S1_at | -1.65 | 0.0035 | FAM134B | NM_019000.3 | Homo sapiens family with sequence similarity 134; member B (FAM134B); transcript variant 2; mRNA |
| Ssc.15774.1.S1_at | -1.66 | 0.0293 | OPRM1 | NM_000914.2 | Homo sapiens opioid receptor; mu 1 (OPRM1); transcript variant MOR-1; mRNA |
| Ssc.16132.1.A1_at | -1.66 | 0.0043 | COL8A1 | NM_001850.3 | Homo sapiens collagen; type VIII; alpha 1 (COL8A1); transcript variant 1; mRNA |
| Ssc.30097.1.A1_at | -1.67 | 0.0059 | CDC42BPA | NM_014826.4 | Homo sapiens CDC42 binding protein kinase alpha (DMPK-like) (CDC42BPA); transcript variant A; mRNA |
| Ssc.10994.1.S1_at | -1.67 | 0.0482 | ASPM | NM_018136.4 | Homo sapiens asp (abnormal spindle) homolog; microcephaly associated (Drosophila) (ASPM); mRNA |
| Ssc.11579.1.A1_at | -1.70 | 0.0317 | GJA10 | NM_032602.1 | Homo sapiens gap junction protein; alpha 10; 62kDa (GJA10); mRNA >gi|14009610|gb|AF296766.1|AF296766 Homo sapiens connexin 62 mRNA; complete cds |
| Ssc.6932.1.A1_at | -1.71 | 0.0423 | DPP10 | NM_020868.2 | Homo sapiens dipeptidyl-peptidase 10 (DPP10); transcript variant 1; mRNA |
| Ssc.7372.1.A1_at | -1.71 | 0.0163 | GPX6 | NM_182701.1 | Homo sapiens glutathione peroxidase 6 (olfactory) (GPX6); mRNA >gi|32492912|gb|AY324826.1| Homo sapiens glutathione peroxidase 6 mRNA; complete cds |
| Ssc.16003.1.S1_at | -1.71 | 0.0172 | SP1 | NM_138473.2 | Homo sapiens Sp1 transcription factor (SP1); mRNA |
| Ssc.11746.1.A1_at | -1.72 | 0.0466 | MST150 | NM_032947.3 | Homo sapiens MSTP150 (MST150); mRNA |
| Ssc.14512.1.S1_at | -1.75 | 0.0332 | MAN1A1 | NM_005907.2 | Homo sapiens mannosidase; alpha; class 1A; member 1 (MAN1A1); mRNA |
| Ssc.18830.1.A1_at | -1.76 | 0.0136 | LRRC67 | NM_001013626.2 | Homo sapiens leucine rich repeat containing 67 (LRRC67); mRNA |
| Ssc.27045.1.A1_at | -1.77 | 0.0044 | IDI1 | NM_004508.2 | Homo sapiens isopentenyl-diphosphate delta isomerase 1 (IDI1); mRNA |
| Ssc.13876.1.S1_at | -1.77 | 0.0227 | NEK2 | NM_002497.2 | Homo sapiens NIMA (never in mitosis gene a)-related kinase 2 (NEK2); mRNA |
| Ssc.14513.1.S1_at | -1.78 | 0.0170 | GPX5 | NM_001509.2 | Homo sapiens glutathione peroxidase 5 (epididymal androgen-related protein) (GPX5); transcript variant 1; mRNA |
| Ssc.5104.1.S1_at | -1.79 | 0.0011 | NUPR1 | NM_012385.2 | Homo sapiens nuclear protein 1 (NUPR1); transcript variant 2; mRNA |
| Ssc.5047.1.A1_at | -1.79 | 0.0465 | LYVE1 | NM_006691.3 | Homo sapiens lymphatic vessel endothelial hyaluronan receptor 1 (LYVE1); mRNA |
| Ssc.16102.1.S1_at | -1.80 | 0.0137 | CRISP1 | NM_170609.1 | Homo sapiens cysteine-rich secretory protein 1 (CRISP1); transcript variant 2; mRNA |
| Ssc.3394.3.A1_at | -1.81 | 0.0000 | CDR2 | NM_001802.1 | Homo sapiens cerebellar degeneration-related protein 2; 62kDa (CDR2); mRNA |
| Ssc.25243.1.S1_at | -1.81 | 0.0220 | EMILIN2 | NM_032048.2 | Homo sapiens elastin microfibril interfacer 2 (EMILIN2); mRNA |
| Ssc.11756.2.S1_at | -1.86 | 0.0067 | CDC2L6 | NM_015076.3 | Homo sapiens cell division cycle 2-like 6 (CDK8-like) (CDC2L6); mRNA |
| Ssc.29615.1.A1_at | -1.87 | 0.0053 | UNC5C | NM_003728.2 | Homo sapiens unc-5 homolog C (C. elegans) (UNC5C); mRNA |
| Ssc.14419.1.S1_at | -1.89 | 0.0376 | DLX6 | NM_005222.2 | Homo sapiens distal-less homeobox 6 (DLX6); mRNA |
| Ssc.7195.2.S1_at | -1.90 | 0.0139 | BUB1 | NM_004336.3 | Homo sapiens budding uninhibited by benzimidazoles 1 homolog (yeast) (BUB1); mRNA |
| Ssc.17335.1.S1_at | -1.91 | 0.0096 | SLC17A1 | NM_005074.3 | Homo sapiens solute carrier family 17 (sodium phosphate); member 1 (SLC17A1); mRNA |
| Ssc.14537.1.S1_at | -1.91 | 0.0160 | SLC22A2 | NM_003058.2 | Homo sapiens solute carrier family 22 (organic cation transporter); member 2 (SLC22A2); mRNA |
| Ssc.3960.2.S1_at | -1.96 | 0.0140 | CDKL2 | NM_003948.3 | Homo sapiens cyclin-dependent kinase-like 2 (CDC2-related kinase) (CDKL2); mRNA |
| Ssc.19222.1.A1_at | -1.97 | 0.0041 | SH3GL3 | NM_003027.2 | Homo sapiens SH3-domain GRB2-like 3 (SH3GL3); mRNA >gi|2921413|gb|AF036271.1|AF036271 Homo sapiens EEN-B2-L3 mRNA; complete cds |
| Ssc.23997.1.S1_at | -1.98 | 0.0037 | CNTN1 | NM_175038.1 | Homo sapiens contactin 1 (CNTN1); transcript variant 2; mRNA |
| Ssc.26308.1.S1_at | -1.99 | 0.0011 | PROCR | NM_006404.3 | Homo sapiens protein C receptor; endothelial (EPCR) (PROCR); mRNA |
| Ssc.955.1.S1_at | -2.00 | 0.0028 | CYP2C19 | NM_000769.1 | Homo sapiens cytochrome P450; family 2; subfamily C; polypeptide 19 (CYP2C19); mRNA |
| Ssc.18789.1.A1_at | -2.01 | 0.0041 | C2orf81 | XM_942034.3 | PREDICTED: Homo sapiens similar to hCG40743 (LOC388963); mRNA |
| Ssc.27096.1.A1_at | -2.01 | 0.0067 | DPY19L2 | NM_173812.4 | Homo sapiens dpy-19-like 2 (C. elegans) (DPY19L2); mRNA |
| Ssc.429.1.A1_at | -2.01 | 0.0463 | S100G | NM_004057.2 | Homo sapiens S100 calcium binding protein G (S100G); mRNA |
| Ssc.10185.1.S1_at | -2.02 | 0.0465 | RSF1 | NM_016578.3 | Homo sapiens remodeling and spacing factor 1 (RSF1); mRNA |
| Ssc.22049.1.S1_at | -2.03 | 0.0148 | QSOX1 | NM_001004128.2 | Homo sapiens quiescin Q6 sulfhydryl oxidase 1 (QSOX1); transcript variant 2; mRNA |
| Ssc.10059.1.A1_at | -2.04 | 0.0171 | HOXA13 | NM_000522.4 | Homo sapiens homeobox A13 (HOXA13); mRNA |
| Ssc.5126.1.A1_at | -2.06 | 0.0068 | DLGAP1 | NM_004746.2 | Homo sapiens discs; large (Drosophila) homolog-associated protein 1 (DLGAP1); transcript variant alpha; mRNA >gi|2588977|dbj|AB000277.1| Homo sapiens mRNA for DAP-1 alpha; complete cds |
| Ssc.27342.1.S1_at | -2.10 | 0.0231 | ONECUT2 | NM_004852.2 | Homo sapiens one cut homeobox 2 (ONECUT2); mRNA |
| Ssc.24855.1.S1_at | -2.12 | 0.0063 | NRXN3 | NM_001105250.1 | Homo sapiens neurexin 3 (NRXN3); transcript variant 3; mRNA |
| Ssc.15433.1.S1_at | -2.18 | 0.0140 | KIF5C | NM_004522.1 | Homo sapiens kinesin family member 5C (KIF5C); mRNA >gi|3043585|dbj|AB011103.1| Homo sapiens mRNA for KIAA0531 protein; partial cds |
| Ssc.24076.1.A1_at | -2.19 | 0.0155 | SFT2D2 | NM_199344.2 | Homo sapiens SFT2 domain containing 2 (SFT2D2); mRNA |
| Ssc.8980.1.A1_at | -2.20 | 0.0000 | ANGPTL4 | NM_139314.1 | Homo sapiens angiopoietin-like 4 (ANGPTL4); transcript variant 1; mRNA |
| Ssc.17345.1.S1_at | -2.21 | 0.0170 | ANGPTL4 | NM_139314.1 | Homo sapiens angiopoietin-like 4 (ANGPTL4); transcript variant 1; mRNA |
| Ssc.4255.1.S1_at | -2.23 | 0.0028 | HAPLN1 | NM_001884.3 | Homo sapiens hyaluronan and proteoglycan link protein 1 (HAPLN1); mRNA |
| Ssc.19038.1.A1_at | -2.32 | 0.0475 | VPS53 | NM_018289.3 | Homo sapiens vacuolar protein sorting 53 homolog (S. cerevisiae) (VPS53); transcript variant 2; mRNA |
| Ssc.25475.1.S1_at | -2.37 | 0.0059 | GRIA3 | NM_000828.4 | Homo sapiens glutamate receptor; ionotrophic; AMPA 3 (GRIA3); transcript variant 2; mRNA |
| Ssc.15982.1.S1_at | -2.41 | 0.0028 | SLC9A5 | NM_004594.2 | Homo sapiens solute carrier family 9 (sodium/hydrogen exchanger); member 5 (SLC9A5); mRNA |
| Ssc.4289.1.S1_at | -2.43 | 0.0084 | LRP2 | NM_004525.2 | Homo sapiens low density lipoprotein-related protein 2 (LRP2); mRNA |
| Ssc.30290.1.S1_at | -2.62 | 0.0009 | SHANK2 | NM_012309.1 | Homo sapiens SH3 and multiple ankyrin repeat domains 2 (SHANK2); transcript variant 1; mRNA |
| Ssc.15327.1.S1_at | -2.67 | 0.0088 | CR2 | NM_001877.3 | Homo sapiens complement component (3d/Epstein Barr virus) receptor 2 (CR2); transcript variant 2; mRNA |
| Ssc.23041.1.S1_at | -2.67 | 0.0007 | LOC651751 | XM_001718996.1 | PREDICTED: Homo sapiens similar to hCG2042722 (LOC651751); mRNA |
| Ssc.28462.1.A1_a_at | -2.78 | 0.0011 | C6orf52 | XM_001718541.1 | PREDICTED: Homo sapiens chromosome 6 open reading frame 52 (C6orf52); mRNA |
| Ssc.28632.3.A1_at | -2.84 | 0.0005 | MBOAT1 | NM_001080480.1 | Homo sapiens membrane bound O-acyltransferase domain containing 1 (MBOAT1); mRNA |
| Ssc.28101.1.A1_at | -2.87 | 0.0019 | AAK1 | NM_014911.3 | Homo sapiens AP2 associated kinase 1 (AAK1); mRNA |
| Ssc.3207.1.S1_at | -2.87 | 0.0086 | CSDC2 | NM_014460.3 | Homo sapiens cold shock domain containing C2; RNA binding (CSDC2); mRNA |
| Ssc.4483.1.A1_at | -2.97 | 0.0009 | SLC19A3 | NM_025243.3 | Homo sapiens solute carrier family 19; member 3 (SLC19A3); mRNA |
| Ssc.8164.1.A1_at | -3.25 | 0.0004 | KCND2 | NM_012281.2 | Homo sapiens potassium voltage-gated channel; Shal-related subfamily; member 2 (KCND2); mRNA |
| Ssc.3937.1.S1_at | -6.29 | 0.0001 | STMN2 | NM_007029.2 | Homo sapiens stathmin-like 2 (STMN2); mRNA >gi|33873514|gb|BC006302.2| Homo sapiens stathmin-like 2; mRNA (cDNA clone MGC:12784 IMAGE:4100671); complete cds |

^#^Positive fold changes indicate that the low protein (LP) diet resulted in higher expression than the high protein (HP) diet and the number represents [expression LP / expression HP]. Negative fold changes indicate that the LP diet resulted in lower expression than the HP diet and the number represents {-[expression HP / expression LP]}.

**Supplementary Table 2- Primer details for reference, differentially expressed** (DE) and non-changing genes

| **Gene category** | **Gene symbol** | **GenBank accession no. (porcine)** | **Sequence (5’ to 3’)** | **Product size (bp)** |
| --- | --- | --- | --- | --- |
| Reference genes | B2M | NM_213978 | FWD: AAACGGAAAGCCAAATTACC  REV: ATCCACAGCGTTAGGAGTGA | 178 |
|  | TBP | DQ845176 | FWD: TTAATGGTGGTGTTGTGGACGGC  REV: CCAAATAGCAGCACAGTACGAGCAA | 168 |
|  | RPL4 | DQ845178 | FWD: AGAGATCCAAAGAGCCCTCCGC  REV: GCCTGGCGAAGAATGGTGTTTC | 144 |
| Differentially expressed genes | MPHOSPH6 | Human acc no: NM_005792.2 | FWD: ATGGGATTCATCAGGAATGG  REV: AGGTCAGTGACTGGGAGAGC | 124 |
|  | UBE2CBP | AK236545.1 | FWD: CCAGCCACAGATTGAGAACA  REV: CTTCACAGCCTTGGCTTTTC | 108 |
|  | LTBR | NM_001146126.1 | FWD: GCAAAACTCGTTGCACACAT  REV: GCAATATTTTGGCACACGTC | 122 |
|  | SCD | AY487830.1 | FWD: CTATGTGACCCTGGGCAAGT  REV: TCAAAACTGCCCTTTGAGGT | 100 |
|  | BTG2 | EU255256.1 | FWD: GCCCCTGCCTTTTTATAAGC  REV: AGACAGGCCTGCTCAACAGT | 119 |
|  | CBX5 | AY610107.1 | FWD: GACAGATTCCTGTGGCGATT  REV: GATATGCATGCCACGTCAGT | 146 |
|  | ANGPTL4 | AY974561.1 | FWD: TGCAAGATGACCTCAGATGG  REV: CTCCGAAGCCATCCTTGTAG | 103 |
|  | IQGAP2 | Human acc no: NM_006633.2 | FWD: AAGTGCACTACTGCCTCGTG  REV: AAATTGTGAGGGGTGGAGAT | 108 |
|  | PROCR-like | AY820762.1 | FWD: CTGGCAAGGGAAGATCTCAG  REV: TCTTCTCCCCTCCCTCAAAT | 138 |
|  | SATB2 | LOC100155877 | FWD: GCATCCGCATTTGGTATCTT  REV: ACAGGCTAAAATGCCCACAG | 140 |
| Non-changing genes | RAP1 | XM_001928649.1 | FWD: TTTCCCCTCAAACTCATTGC  REV: AGACCCATTTTTCCAAAGCA | 139 |
|  | MRPS6 | Human acc no: NM_032476.3 | FWD: TGGCTTGCTTAGAGCCACTT  REV: CCAAAGGGCGTTAGTGTTGT | 117 |
|  | PSMD1 | XM_001925902.1 | FWD: AATGCCTGTGAGGAAATTCG  REV: CCAAGTCGTGTGAAAGAGCA | 135 |

FWD: forward primer; REV: reverse primer; bp: base pairs.
